# Dental pulp mesenchymal stem cell-derived exosomes inhibit neuroinflammation and microglial pyroptosis in subarachnoid hemorrhage via the miRNA-197-3p/FOXO3 axis

**DOI:** 10.1186/s12951-024-02708-w

**Published:** 2024-07-19

**Authors:** Xin Liang, Yan Miao, Xin Tong, Jigang Chen, Hongyi Liu, Zilong He, Aihua Liu, Zhiqiang Hu

**Affiliations:** 1 Department of Neurosurgery, Affiliated Beijing Shijitan Hospital, Capital Medical University, Beijing, 100038 China; 2 Department of Neurology, The Third Xiangya Hospital, Central South University, Changsha, 410013 China; 3 Beijing Neurosurgical Institute, Beijing Tiantan Hospital, Capital Medical University, Beijing, 100070 China; 4 Department of burn and plastic surgery, Beijing Children’s Hospital, Capital Medical University, Beijing, 100045 China; 5 School of Biomedical Engineering, Capital Medical University, Beijing, 100069 China; 6 Department of Neurosurgery, The Third Xiangya Hospital, Central South University, Changsha, 410013 China; 7 Department of Interventional Neuroradiology, Beijing Tiantan Hospital, Capital Medical University, Beijing, 100070 China; 8 China National Clinical Research Centre for Neurological Diseases, Beijing, 100070 China; 9 Department of Neurosurgery, Affiliated Beijing Jishuitan Hospital, Capital Medical University, Beijing, China; 10 Cerebrovascular Disease Department, Neurological Disease Center, Beijing Anzhen Hospital, Capital Medical University, Beijing, China

**Keywords:** Dental pulp stem cell, Exosomes, Pyroptosis, Subarachnoid hemorrhage, miRNA

## Abstract

**Background:**

Subarachnoid hemorrhage (SAH) is a severe stroke subtype that lacks effective treatment. Exosomes derived from human dental pulp stem cells (DPSCs) are a promising acellular therapeutic strategy for neurological diseases. However, the therapeutic effects of DPSC-derived exosomes (DPSC-Exos) on SAH remain unknown. In this study, we investigated the therapeutic effects and mechanisms of action of DPSC-Exos in SAH.

**Materials and methods:**

SAH was established using 120 male Sprague-Dawley rats. One hour after SAH induction, DPSC-Exos were administered via tail vein injection. To investigate the effect of DPSC-Exos, SAH grading, short-term and long-term neurobehavioral assessments, brain water content, western blot (WB), immunofluorescence staining, Nissl staining, and HE staining were performed. The role of miR-197-3p/FOXO3 in regulating pyroptosis was demonstrated through miRNA sequencing, bioinformatics analysis, and rescue experiments. The SAH model in vitro was established by stimulating BV2 cells with hemoglobin (Hb) and the underlying mechanism of DPSC-Exos was investigated through WB and Hoechst/PI staining.

**Results:**

The expressions of pro-inflammatory cytokines (IL-1β, IL-6, and TNF-α) were increased after SAH. DPSC-Exos alleviated brain edema and neuroinflammation by inhibiting the expression of FOXO3 and reducing NLRP3 inflammasome activation, leading to improved neurobehavioral functions at 24 h after SAH. In vitro, the expression of the NLRP3 inflammasome components (NLRP3 and caspase1-p20), GSDMD-N, and IL-18 was inhibited in BV2 cells pretreated with DPSC-Exos. Importantly, DPSC-Exos overexpressing *miR-197-3p* had a more obvious protective effect than those from NC-transfected DPSCs, while those from DPSCs transfected with the *miR-197-3p* inhibitor had a weaker protective effect. Functional studies indicated that *miR-197-3p* bound to the 3ʹ-untranslated region of *FOXO3*, inhibiting its transcription. Furthermore, the overexpression of *FOXO3* reversed the protective effects of *miR-197-3p*.

**Conclusions:**

DPSC-Exos inhibited activation of the NLRP3 inflammasome and related cytokine release via the miR-197-3p/FOXO3 pathway, alleviated neuroinflammation, and inhibited microglial pyroptosis. These findings suggest that using DPSC-Exos is a promising therapeutic strategy for SAH.

**Graphical Abstract:**

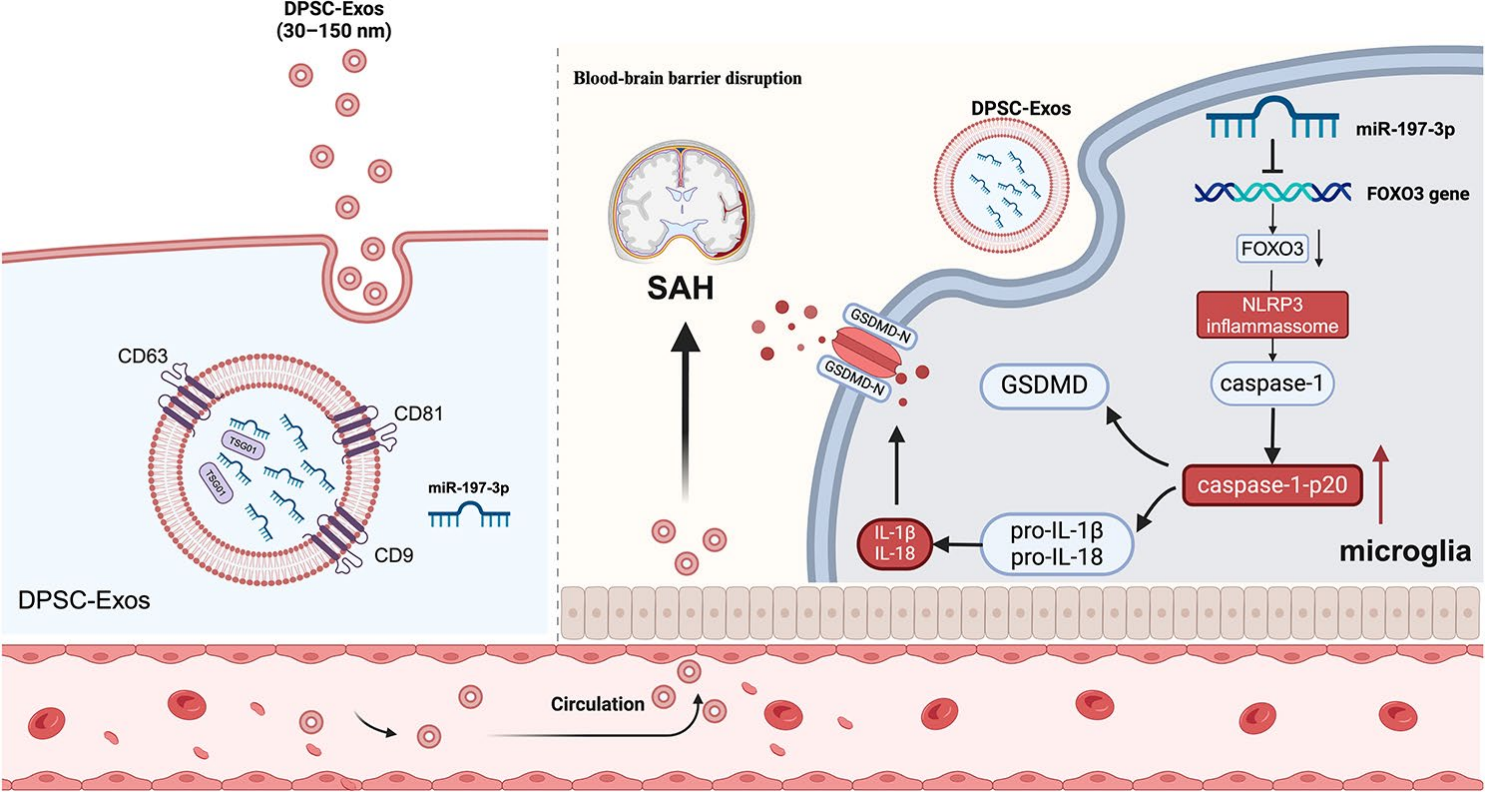

**Supplementary Information:**

The online version contains supplementary material available at 10.1186/s12951-024-02708-w.

## Background

Subarachnoid hemorrhage (SAH) caused by an aneurysm is a severe subtype of stroke that accounts for approximately 5% of all stroke cases. Compared with other types of strokes, SAH is characterized by a younger age of onset as well as higher incidence and mortality rates [1–3]. Early brain injury (EBI) within the first 72 h after SAH onset is a primary factor leading to poor prognosis [4, 5]. Researchers have elucidated the potential pathological mechanisms related to EBI, including oxidative stress damage, blood-brain barrier dysfunction, neuronal apoptosis, and neuroinflammation. Notably, SAH-related neuroinflammation plays a key role in EBI and is considered a critical target for potential interventions [6]. Therefore, neuroinflammation interventions during EBI in SAH can lead to improved neurobehavioral outcomes. Microglia are key inflammatory cells in the central nervous system and can exhibit either a pro-inflammatory M1 or anti-inflammatory M2 phenotype, a process known as microglial polarization [7, 8]. The number of microglia increases after stroke, and when these cells are activated for the M1 phenotype they release pro-inflammatory cytokines, such as TNF-α, IL-1β, and IL-6, further causing tissue damage and neurological deficits [9–12]. Conversely, polarization of microglia towards an anti-inflammatory phenotype can attenuate neuroinflammation and tissue damage in EBI following SAH [13, 14].

Pyroptosis is a form of programmed cell death. In the central nervous system, microglia pyroptosis occurs [15]. When microglia are subjected to infection or other stimuli, activation of the NLRP3 inflammasome results in the assembly of the downstream adaptor protein, ASC, into an inflammasome complex, which leads to the activation of pro-caspase-1 [16]. The activation of caspase-1 mediates the cleavage of pro-IL1β and pro-IL-18, resulting in an increased secretion of the mature forms of IL-1β and IL-18 [17]. Additionally, caspase-1 directly cleaves its substrate, gasdermin D (GSDMD), and converts it into its active phenotype, GSDMD-N. Nonselective pores in GSDMD-N on the cell membrane led to cell infiltration, swelling, and rupture, causing the release of intracellular inflammatory factors and pyroptosis [18]. In Alzheimer’s disease, microglia regulate NLRP3-ASC inflammasomes and associated inflammatory changes through Tau [19]. Activation of NLRP3 in microglia exacerbates neuroinflammation and neurodegeneration in Parkinson’s disease regulated by MPTP [20]. In animal models of Parkinson’s disease [21], depression [15], inflammatory pain [22], and mechanisms, such as ubiquitination, autophagy, and mitophagy, inhibit NLRP3-mediated inflammasome activation, thereby reducing neuroinflammation and oxidative reactions and preserving neuronal activity. Furthermore, microglia pyroptosis mediated by NLRP3 inflammasomes are involved in EBI following SAH [23]. Inhibiting the activation of NLRP3 and related pathways can reduce inflammation [24], inhibit delayed cerebral vasospasm [25], and reduce M1 polarization of microglia [26]. Although antimicroglial pyroptosis therapy seems promising for SAH, most pyroptosis inhibitors are delivered to the brain via local stereotactic injection, which can cause damage to the brain tissue. However, intravenous administration faces challenges in penetrating the blood-brain barrier.

Recently, mesenchymal stem cells (MSCs) became a focus of research. MSCs are known for their ability to inhibit neuroinflammation, promote tissue regeneration, and stimulate angiogenesis. Their primary mechanism of action involves exerting their biological functions via paracrine mechanisms [27]. Compared with the commonly used bone marrow-derived (BMSCs) and adipose-derived MSCs (ADSCs), DPSCs secrete more neurotrophic factors and possess stronger regenerative potential [28, 29]. Moreover, obtaining DPSCs is safer and less painful than obtaining BMSCs or ADSCs. Thus, DPCSs are ideal cell sources for stem cell therapy. However, there are some limitations to the use of MSCs in the treatment of SAH. First, the cultivation and preservation of MSCs are challenging, with low survival rates. Second, as allogeneic stem cells, MSCs may induce immune rejection. Third, MSCs have an inherent risk of tumorigenesis. Exosomes, factors secreted by MSCs, have shown substantial therapeutic potential in diseases, such as Parkinson’s disease, cerebral infarction, and myocardial infarction [30–32]. Exosomes are extracellular vesicles, 30–150 nm in diameter and play a crucial role in intercellular communication by transferring DNA, RNA, proteins, and lipids [33]. MSC-derived exosomes alleviate neuroinflammation and autophagy in EBI after SAH, providing neuroprotection [34]. Conditioned media from DPSCs can reduce the expression of inflammatory factors in AQP4, inhibit the M1 polarization of microglia, alleviate cerebral edema, and improve microcirculation following SAH [35]. However, its effector factors and mechanisms of action remain unclear. Therefore, we hypothesized that exosomes derived from DPSCs alleviate post-SAH EBI by inhibiting microglial pyroptosis.

In the present study, we aimed to demonstrate the therapeutic potential of DPSC-derived exosomes (DPSC-Exos) in the treatment of SAH, particularly through the inhibition of microglial pyroptosis, thereby suppressing neuroinflammation and attenuating neurological deficits. Our results showed that DPSC-Exos can inhibit pyroptosis in microglia following SAH, contributing to improvements in neurological deficits. miR-197-3p enrichment in exosomes mediates this effect by negatively regulating FOXO3, thereby alleviating post-SAH EBI.

## Methods

### SAH cell model

BV2, a microglial cell line, was purchased from Seville Company (Wuhan, China). BV2 was cultured in a complete medium at 37 °C in 5% CO_2_. The complete medium contained 10% fetal bovine serum (FBS, 10,099,141, Gibco, USA), 100 U/mL penicillin, 100 U/mL streptomycin (15,070,063, Gibco, USA), and high-glucose Dulbecco’s Modified Eagle Medium (DMEM, 8,121,513, Gibco, USA), and was replaced every 2 d.

The SAH in vitro model was established by adding Hemoglobin (Hb; 10 µM, H7379, Sigma-Aldrich, USA) to the completed medium of BV2 cells. Untreated cells were used as controls. To further elucidate the impact of DPSC-Exos on BV2 induced by Hb, DPSC-Exos (10 µg/mL) and BV2 induced by Hb were co-incubated for 24 h.

### Isolation and culture of DPSCs

DPSC isolation was quantified according to the method published by Razieh [36]. Human third molars were obtained from adults (18–26 years of age). at the Department of Stomatology, Beijing Tiantan Hospital (Beijing, China). The clinical study was approved by the Institutional Review Board of Beijing Tiantan Hospital, Capital Medical University, Beijing, China. The teeth were aseptically processed to remove the surface tissues and sectioned at the dentin-enamel junction using a sterilized dental bur to expose the pulp chamber. Pulp tissue was extracted and cut into small pieces. The shredded pulp tissue was placed in an enzyme solution containing 3 mg/mL type I collagenase (17,100,017, Gibco, USA) and 4 mg/mL dispase II (04942078001, Sigma-Aldrich, USA) and incubated at 37 °C for 1 h with vortexing every 15 min. The enzymatic reaction was terminated using complete culture medium. The digested pulp suspension was filtered through a 70-µm cell strainer to obtain a single-cell suspension. After centrifugation at 300 g or 5 min, the cells were resuspended and cultured in complete medium at 37 °C with 5% CO_2_. The medium was changed on day 7 and then every 2 d. DPSCs from passages 3 to 5 were used for subsequent experiments.

DPSC surface markers were validated using a BD CytoFLEX S flow cytometer (BD Biosciences, USA). In brief, cells were digested, resuspended in phosphate-buffered saline (PBS), and then incubated in the dark at 4 °C for 30 min with the following antibodies: PE Anti-Human CD105 Antibody (E-AB-F1243D, Elabscience, China), PE Anti-Human CD90 Antibody (E-AB-F1167D, Elabscience, China), PE Anti-Human CD45 Antibody (E-AB-F1039D, Elabscience, China), PE Anti-Human HLA-DR Antibody (E-AB-F1111D, Elabscience, China), FITC Anti-Human CD34 Antibody (E-AB-F1143C, Elabscience, China), PE anti-Human CD14 (301,805, BioLegend, USA), and PE anti-human CD73 (344,004, BioLegend, China). The percentage of stained cells was quantified using FlowJo software (version 10.9).

DPSCs (2.5 × 10^5^ cells/well) were seeded in a six-well plate. After 21–28 d of culture in adipogenic differentiation medium (complete medium supplemented with 10 µg/mL insulin, 0.5 mM 3-isobutyl-1-methylxanthine, 200 µM indomethacin, and 1 µM dexamethasone) and osteogenic differentiation medium (complete medium supplemented with 10 mM β-glycerophosphate, 50 µM vitamin C, and 0.1 µM dexamethasone), the multipotent differentiation ability of DPSCs was assessed using Oil Red O staining and Alizarin Red S staining.

### DPSC-Exos isolation and characterization

DPSC cultures at passages 3–5 was maintained until 90% confluence was achieved. The bottom of DPSC 175 cm^2^-culture flasks was washed three times with PBS, and the medium was replaced with 20 mL of DMEM containing 10% extracellular vesicle-free FBS (EXO-FBS-50 A-1, System Biosciences, USA). After 48 h of further cultivation, the culture supernatant from DPSCs was collected and stored at -80 °C.

The collected culture medium was sequentially centrifuged at 4 °C (300 × g, 15 min; 3000 × g, 10 min; and 12,000 × g, 30 min) to remove cells, debris, and vesicles. The clarified supernatant was then filtered using a 0.22-µm syringe filter (SLGV033R, Sigma-Aldrich, USA). The filtered supernatant was subjected to two rounds of 4 °C ultracentrifugation at 100,000 × g for 90 min each, followed by resuspension of the remaining pellet in PBS. The remaining deposit was resuspended in PBS and stored at -80 °C.

The concentration of DPSC-Exos was determined using the Pierce™ BCA protein assay kit (23,227, Thermo Scientific, USA). To assess the size distribution of exosomes, samples diluted in PBS were subjected to nanoparticle tracking analysis (NTA) using a ZetaView system (Particle Metrix, Germany). Transmission electron microscopy (TEM, HITACHI, Japan) was used to observe the morphology of the exosomes. To detect surface markers on exosomes, western blotting (WB) was performed using the following antibodies: Anti-CD9 (ab223052, Abcam, UK), Anti-CD63 (ab134045, Abcam, UK), anti-Alix (ab186429, Abcam, UK), Anti-TSG101 (ab125011, Abcam, UK).

### Animals

#### SAH rat model

Adult male Sprague-Dawley rats (*n* = 120, weight = 280–320 g, Vital River, China) that met specific pathogen-free standards were used in this study. All animals were housed in a controlled environment with humidity maintained at 60 ± 5% and temperature at 25 ± 1 °C. They were subjected to a 12-h light-dark cycle and had access to adequate food and water.

The experimental SAH model via endovascular perforation was based on a previously published study [23]. Briefly, the rats were anesthetized with an intraperitoneal injection of pentobarbital (40 mg/kg body weight). After exposing the right common carotid artery and its branches, a 4 − 0 monofilament nylon suture was inserted into the stretched external carotid artery and advanced to the bifurcation of the anterior and middle cerebral arteries. The rats in the sham surgery group underwent the same surgical procedure without puncturing. After surgery, the animals were transferred to a heated cage at 37.5 °C for recovery, with respiratory rate, heart rate, and skin color monitored every 15 min until normal recovery. This study was approved by the Ethics Committee of Tiantan Hospital (Ethics number: 202,201,012), and all experiments and procedures adhered to animal ethical standards.

### Experimental design

The animals were randomly assigned to five independent experiments. All the animals were randomized upon entry into the experiments. During the analysis, the animals were randomly assigned to treatment groups. For the immunostaining imaging experiments, the researcher was blinded to the selection of glass slides used for microscopic imaging.

#### Experimental design 1

To assess the effect of DPSC-Exos on EBI following SAH, 18 rats were randomly divided into three groups (*n* = 6 per group): Sham, SAH + PBS, and SAH + Exos. Within 1 h post-SAH, rats were subjected to a gradual caudal vein administration of 100 µg of exosomes. An equivalent volume of PBS was administered to the control group. At 24 h post-SAH, the rats in all groups were evaluated for SAH grade, neurological behavioral performance (modified Garcia score) and brain water content.

#### Experimental design 2

To investigate the effect of DPSC-Exos on microglia/macrophage activation 24 h post-SAH, 30 rats were randomly divided into three groups (*n* = 10 per group): Sham, SAH + PBS, and SAH + Exos. Fluorescent staining with CD68 was performed on calcium-binding adapter molecule 1 (Iba-1) positive microglial cells (*n* = 4 per group), and CD68-positive cells in the perihematomal area were counted 24 h post-SAH. WB was conducted to assess neuroinflammation in the ipsilateral (right) hemisphere at 24 h post-SAH. The brain tissue samples from these three groups were similar to those used in Experiment 5.

### Experimental design 3

To assess the distribution of exosomes in the rat brain, DPSC-Exos labeled with PKH67 (MINI67-1KT; Sigma-Aldrich, USA) were observed. Four rats were randomly assigned to one of the two groups: SAH + PBS and SAH + Exos (*n* = 2 per group). At 24 h post-SAH, immunofluorescence staining was conducted to evaluate PKH67-labeled exosomes, along with neuronal nuclei (NeuN), glial fibrillary acidic protein (GFAP), and Iba-1.

### Experimental design 4

To evaluate the long-term effects of DPSC-Exos post-SAH, rats were randomly divided into three groups: Sham, SAH + PBS, and SAH + Exos. The Morris water maze test was used to evaluate the cognitive changes in rats from days 23 to 27 post-SAH (*n* = 10 per group). Nissl and hematoxylin and eosin (HE) staining were performed to assess the long-term neuronal degeneration and tissue damage on the 28th day post-SAH.

### Experimental design 5

To explore the molecular mechanisms underlying the anti-inflammatory effects of DPSC-Exos after SAH, 18 rats were randomly divided into three groups (*n* = 6 per group). The right hemisphere of the brain, corresponding to the side of the hemorrhage, was extracted for protein imprinting experiments to assess protein expression in the pathway 24 h post-SAH.

### SAH grading

According to a previously established method [37], two researchers independently graded the severity of SAH bleeding in rats on a scale of 0–18 points. In summary, following the euthanasia of rats, we assessed the extent of bleeding in different regions of the basal brain surface. The criteria were as follows: no blood clots, no points; a small amount of blood clots, one point; a moderate amount of blood clots visible in the arteries, two points; and blood clots covering all arteries, three points. Rats with an SAH bleeding severity grade of less than eight points were excluded from the experiment.

### Exosomes tracing

DPSC-Exos were stained with PKH67 according to the manufacturer’s instructions (MINI67-1KT, Sigma-Aldrich). In brief, 500 µg of DPSC-Exos were diluted with 4 µL PKH67 in 1 mL staining buffer for 15 min. Subsequently, 1 mL of termination buffer containing 2% bovine serum albumin was added and the labeled exosomes were washed by centrifugation at 100,000×g for 2 h. The labeled exosomes were resuspended in PBS prior to administration. As a negative control, 4 µL of PKH67 dye was added to 1 mL staining buffer and incubated for 15 min, followed by incubation with an equal volume of PBS. The resulting pellet, obtained by high-speed centrifugation and containing a small amount of free dye, was injected into SAH rats as a control.

### Neurological scoring

At 24 h post-SAH, we assessed neurological behavior using the modified Garcia scoring system [38]. This system comprises six tests: spontaneous activity (0–3 points), response to lateral touches (1–3 points), whisker touches (1–3 points), limb symmetry (0–3 points), forelimb extension (0–3 points), and climbing (0–3 points). The total scores for these tests ranged from 3 to 18 [37]. Higher scores indicated better neurological behavior.

### Morris water maze

As previously established, the Morris water maze was employed to assess spatial learning capacity and memory in animals [39]. The rats were tested on the days post-SAH. Briefly, rats were placed on the platform for 5 s on the first day. On the subsequent to the spatial learning tests, the animals were randomly placed in different starting positions and tasked with finding the platform submerged in the water.

The time allotted to each animal to locate the platform was 60 s. If the animal failed to locate the platform within 60 s, it was placed on the platform and allowed to remain there for 5 s. The probe trial phase was conducted on day 27 post-SAH, involving the removal of the submerged platform and prompting animals to explore the area where the platform used to be. Animal activity and swimming patterns were recorded via video, and computer tracking systems (EthoVision XT, Noldus, Netherlands) were used to quantify swimming distances, escape latencies, and swim paths.

### Brain water content

As previously established, the brain water content was assessed using the wet-dry method to evaluate the degree of cerebral edema [40]. At 24 h post-SAH, the rats were euthanized, and the entire brain was removed and weighed to obtain the wet weight. Subsequently, the brains were dried in an oven at 95 °C for 72 h and weighed to obtain dry weight. The percentage of brain water content was calculated using the following formula:

Brain water content = wet weight − dry weight) / wet weight × 100%.

### RNA extraction and qRT-PCR

Total RNA was extracted using the TRIzol reagent (15,596,026, Invitrogen, USA). Reverse transcription of 1 µg RNA into cDNA was performed using a reverse transcription kit (G592, Applied Biological Materials, Canada). Reverse transcription of miRNAs was performed using a stem-loop reverse transcription kit (MR101-01, Vazyme, China). The expression of the target genes was assessed using the RT-PCR performed with the SYBR Green Master Mix (4,913,850,001, Roche, Switzerland) and the QuantStudio™ 3 Real-Time PCR System (Applied Biosystems, USA). The PCR and reverse transcription primers were purchased from Sangon Biotech (Shanghai, China). The primer sequences are listed in Table 1. All data for each sample were collected in triplicates. Standard curves were generated, and the relative quantity of miRNA was normalized to the U6 level, while the relative quantity of mRNA was normalized to the β-actin level using the standard 2^(-ΔΔCt) method for calculations.

### Western blot analysis

WB was conducted following a previously established method [41]. Cells at a confluence of 70–80% were treated under the specified conditions. After washing with PBS, the cells were scraped into an ice-cold RIPA lysis buffer (R0010, Solarbio, Shanghai, China) containing a protease inhibitor (ST505, Beyotime, China) for 30 min. The cell lysates were centrifuged at 14,000 × g for 30 min and the supernatant was collected for subsequent experiments.

For all experiments, the rats were deeply anesthetized 24 h after the SAH and euthanized by transcardial perfusion with ice-cold PBS. The brain tissue was dissected into the ipsilateral and contralateral hemispheres. The ipsilateral hemisphere was homogenized in RIPA lysis buffer (R0010, Solarbio, China) containing a protease inhibitor (ST505, Beyotime, China) and lysed for 30 min. The homogenized tissue was then centrifuged at 14,000 × g for 30 min and the supernatant was collected for subsequent experiments.

The collected supernatant was quantified for protein concentration using the Pierce™ BCA protein assay kit (23,225, Thermo Fisher Scientific, USA). Equal amounts of protein (30–50 µg) were loaded onto 7.5%, 10% or 12.5% SDS-PAGE gels for electrophoresis and subsequently transferred to nitrocellulose membranes using a wet transfer system. The membrane was blocked with 5% skimmed milk at 37 °C for 2 h and then incubated with the following primary antibodies overnight at 4 °C: IL-1β (1:1000, Abcam, UK), IL-6 (1:1000, Proteintech, China), TNF-α (1:1000, Abcam, UK), FOXO3 (1:2000, Proteintech), NLRP3 (1:1000, Abmart, China), IL-18 (1:1000, Abmart, China), caspase-1 (1:1000, Proteintech, China), and GSDMD (1:1000, Proteintech, China). β-actin (1:5000, Cell Signaling Technology, USA) served as the internal control protein. Horseradish peroxidase-conjugated secondary antibodies against goat anti-mouse or rabbit IgG (1:5000, ZSbio, China) were used. An enhanced chemiluminescence reagent (WBAVDCH01, Sigma-Aldrich, USA) was used for visualization. Quantitative analysis was performed using the ImageJ software (ImageJ 1.53, NIH, USA).

### Hematoxylin-eosin and nissl staining

As previously described [42–44], HE and Nissl staining were conducted to observe long-term brain pathological changes and neuronal viability in rats following SAH. Brain tissues were fixed overnight in formalin at 4 °C, washed with PBS, and embedded in paraffin. Coronal sections of 5-µm thickness were obtained from the brain tissue. Subsequently, the sections were stained with hematoxylin and eosin to assess brain tissue damage and 0.5% cresyl violet staining was performed to visualize the Nissl bodies in the neurons. Photomicrographs were captured using an optical microscope (Nikon, Japan).

### Immunofluorescence imaging

After sodium pentobarbital anesthesia, the rats in each group were intracardially perfused with PBS until the liver turned pale, followed by perfusion with 100 mL of 4% paraformaldehyde until their bodies became rigid. The entire brains of the rats were rapidly collected and immersed in 4% paraformaldehyde for 24 h at 4 °C. After sucrose dehydration, the brains were embedded in OCT compound (Sakura, Japan) and rapidly frozen at -80 °C. Subsequently, the brains were sectioned into 5-µm coronal brain slices. The slides were washed three times for 5 min each in PBS and incubated at room temperature for 10 min in 0.3% Triton-100, followed by blocking with 5% donkey serum for 2 h. The slides were then incubated overnight at 4 °C with the following primary antibodies: anti-Iba1 (1:200, Abcam, UK) and CD68 (1:200, Abcam, UK). After incubation with the primary antibody, the samples were washed with PBS and incubated at room temperature for 1 h with the corresponding fluorescently labeled secondary antibodies (1:500, Zsbio, China). Images were captured using a Leica DMi8 microscope and Iba-1/CD68 double-positive cells were identified and counted in three different fields around the cortex on the hemorrhagic side from six random coronal sections per rat. Quantitative analysis of positive cells was performed at 200x magnification (*n* = 4 per group). Quantification of the fluorescence intensity was performed using ImageJ software.

### Hoechst 33,342/PI staining

The treated cells were collected and incubated with Hoechst 33,342 and PI (HY-K1070, MCE, USA) at 4 °C for 20 min, then observed under a fluorescence microscope (Fujifilm, Japan).

### Luciferase reporter assay

Fluorescence reporter gene assays were employed to determine whether the *miR-197-3p* can bind to the3ʹ-untranslated region (3ʹ-UTR) of *FOXO3* mRNA. In brief, the wild-type rat 3ʹ-UTR sequence with the *miR-197-3p* binding site, Rat-FOXO3-WT, was cloned into the pmirGLO luciferase reporter gene vector (Tsingke Biotech, Shanghai, China). Similarly, the mutant rat 3ʹ-UTR sequence with the *miR-197-3p* binding site mutated (Rat-FOXO3-MUT) was cloned into the pmirGLO luciferase reporter gene vector. The pmirGLO luciferase reporter gene vector and *miR-197-3p* mimic or NC mimic were co-transfected for 6 h using Lipofectamine 2000 (11,668,030, Invitrogen, USA). Finally, the cells were collected, and luciferase activity was assessed using a dual-luciferase reporter gene assay kit (E1910, Promega, China).

### miRNA mimic and inhibitor transfection or *FOXO3* overexpression

DPSCs were cultured in 175 cm² cell culture flasks, and when the cell density reached 70-80% confluence, transfection with the miRNA mimic and inhibitor was performed. Following the manufacturer’s instructions, Lipofectamine™ 3000 transfection reagent (L3000075, Thermo Fisher Scientific) was used to transfect the *miR-197-3p* mimic, *miR-197-3p* inhibitor, and their respective negative controls (RiboBio, China) into DPSCs. After 24 h of transfection, cells were used for subsequent experiments. Overexpression of FOXO3 was achieved by integrating the FOXO3 genome (GENECHEM, Shanghai, China) into the BV2 genome using lentivirus virus transfection with the multiplicity of infection (MOI) = 50.

### Statistical analysis

Statistical analyses and graph plots were generated using GraphPad Prism 9 (GraphPad Software, USA). All data were presented as mean ± SD. One-way ANOVA and Tukey’s post hoc analysis were used for multiple mean comparisons. Differences were considered statistically significant at *P* < 0.05.

## Results

### Isolation of DPSCs and DPSC-Exos

**Fig. 1:**
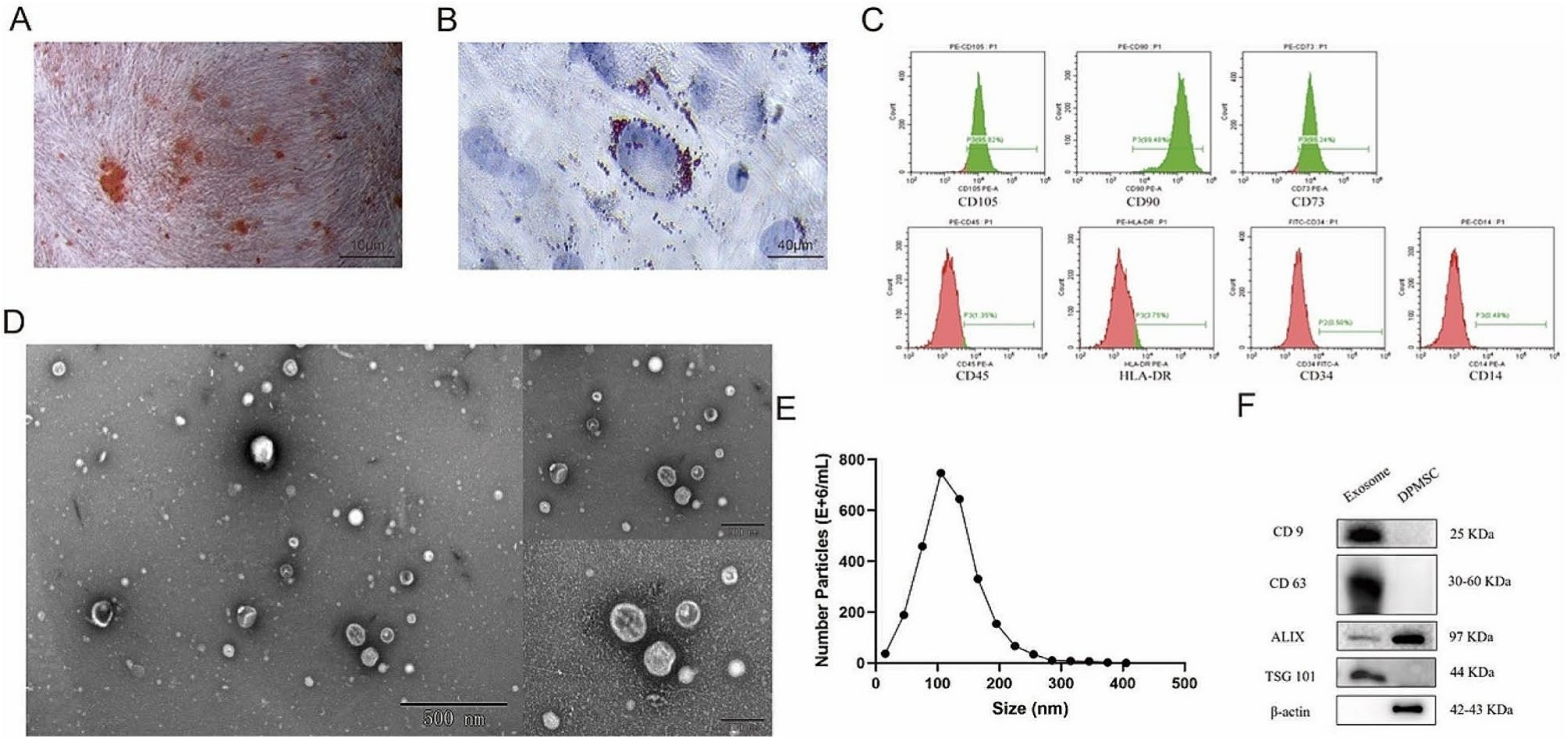
Characterization of DPSCs and DPSC-Exos. (**A**) Alizarin Red staining revealing red calcium nodules in cells. (Scale = 40 μm) (**B**) Oil Red O staining indicating abundant red lipid droplets in the cytoplasm (Scale = 10 μm). (**C**) Flow cytometric analysis of DPSC surface markers CD90, CD105, CD73, and negative markers CD45, HLA-DR, CD34, CD14. (**D**) Representative TEM image of DPSC-Exo (Scale = 500 nm). (**E**) Nanoparticle tracking analysis for the diameter of DPSC-Exos. (**F**) Confirmation of the expression of DPSC-Exo markers CD9, CD63, Alix, TSG101 by WB. DPSC, dental pulp-derived mesenchymal stem cell; DPSC-Exo, DPSC exosome; TEM, transmission electron microscopy; WB, western blotting

The osteogenic and adipogenic differentiation capabilities of the DPSCs obtained from adult third molars were confirmed by Oil Red O, Alizarin Red staining and Alcian blue staining, respectively (Fig. 1A, B, S5). Additionally, flow cytometry was used to identify DPSCs by examining the expression of cell surface antigens in third passage (P3) cells. As shown in Fig. 1C, positive expression of CD105 (95.82%), CD90 (99.48%), and CD73 (95.24%) and negative expression of CD45 (1.35%), HLA-DR (3.75%), CD34 (0.5%), and CD14 (0.48%) were observed, meeting the identification criteria for DPSCs [36].

DPSC-Exos were extracted from the culture medium of primary DPSCs between passages 3 to 5. As shown in Fig. 1, TEM confirmed the typical cup-shaped discoid structure of the exosomes (Fig. 1D). NTA revealed that the average diameter of the DPSC-Exos was 120.2 nm (Fig. 1E). WB was used to evaluate the protein markers of exosomes, using DPSCs as a negative control, which demonstrated the expression levels of CD9, CD63, Alix, and TSG101 (Fig. 1F). Overall, these results confirmed the successful isolation of DPSC-Exos.

### Mortality rate and grading of SAH severity

**Fig. 2:**
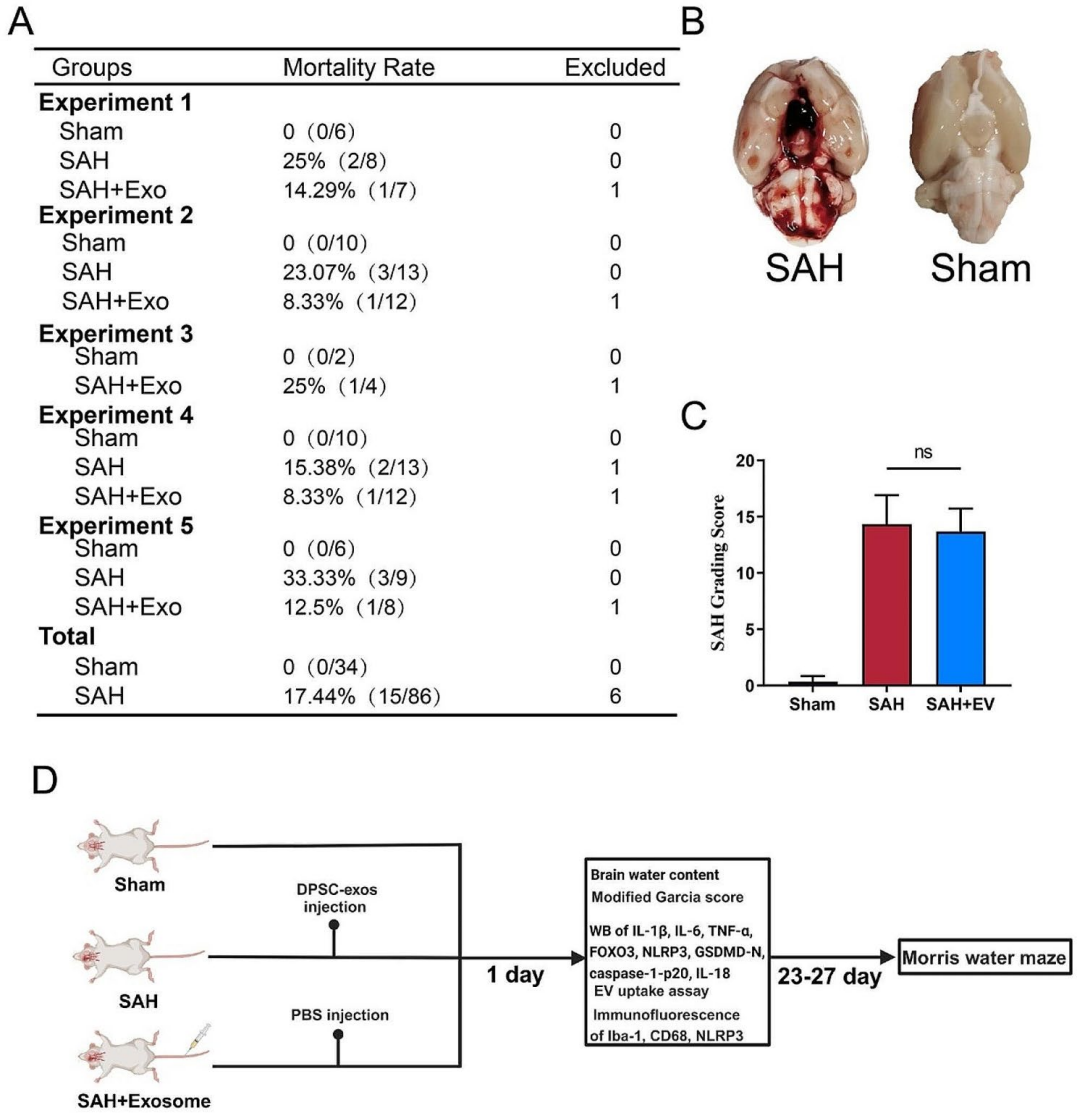
Mortality rate and grading of SAH severity (**A**) Animal usage and mortality rates in all experimental groups. (**B**) Representative image showing subarachnoid hemorrhage clots primarily around Willis circle in rat brains 24 h post-SAH. (**C**) SAH grading scores for all SAH groups. (**D**) Rats were administered PBS or exosomes via tail vein injection 1 h post-SAH, followed by euthanasia and behavioral assessment at specific time points. DPSC, dental pulp-derived mesenchymal stem cell; DPSC-Exo, DPSC exosome; PBS, phosphate-buffered saline; SAH, subarachnoid hemorrhage; WB, western blotting

The intravascular puncture method is a classic animal model of subarachnoid hemorrhage [30] and was employed in our study. Of the 120 rats, 86 underwent SAH modeling through internal carotid artery puncture, with 15 (17.4%) dying within 24 h post-SAH. Six rats were excluded from the study due to a lower degree of basal hemorrhage. No animals in the Sham group died (Fig. 2A). At 24 h post-SAH, photographs of the rat cranial base showed abundant subarachnoid blood clots around the circle of Willis and ventral aspect of the brainstem (Fig. 2B). There was a significant difference in the grading between the Sham and SAH groups (*P* < 0.05). There was no significant difference in the SAH grading scores between the SAH and SAH + Exos groups in each experiment (Figure C). The designed study protocol aimed to investigate the neuroprotective effects of DPSC-Exos in rats (Figure D).

### DPSC-Exos mitigate neurological deficits and brain water content following EBI Post-SAH

**Fig. 3:**
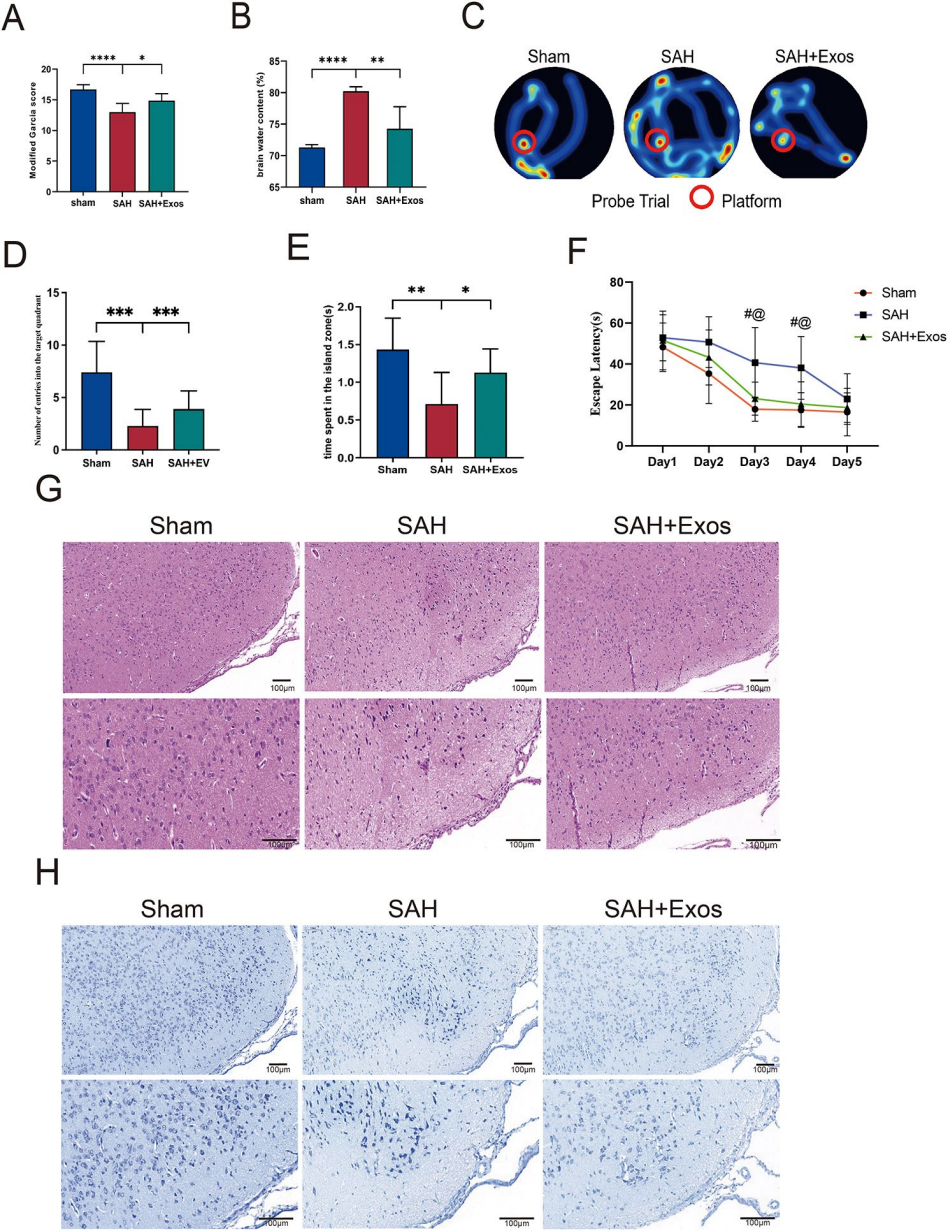
DPSC-Exos mitigate neurological deficits and brain water content following EBI post-SAH. (**A**) DPSC-Exos administration improved neurological behavior as assessed by modified Garcia scores 24 h post-SAH (*n* = 6). (**B**) Measurement of brain water content 24 h post-SAH (*n* = 6). (**C**) Representative tracking images of the swimming paths for each group. (**D**) Recording and analysis of the number of entries into the target quadrant (*n* = 10). (**E**) Recording and analysis of the time spent in the island area during the probe trial (*n* = 10). (**F**) Escape latency in the Morris Water Maze test. (**G**) Representative images of long-term HE staining in rats. (**H**) Representative images of long-term Nissl staining in rats. Data are presented as mean ± standard deviation (SD). (*P < 0.05, **P < 0.01, ***P < 0.001, ****P<0.0001) #*P* < 0.05 vs. Sham group; @*P* < 0.05 vs. SAH + Exos group; DPSC, dental pulp-derived mesenchymal stem cell; DPSC-Exo, DPSC exosome; EBI, early brain injury; SAH, subarachnoid hemorrhage

To evaluate the therapeutic effect of DPSC-Exos, 100ug of Exos were slowly injected into the rats via tail vein 1 h after SAH modeling. Rats in the control group were injected with the same dose of PBS. After 24 h post-SAH, the modified Garcia score, brain water content, and Morris water maze performance was evaluated. The results showed significant neurological impairment in the SAH + PBS group compared with that in the Sham group (Fig. 3A–C). Compared with the SAH + PBS group, the SAH + Exos group achieved higher modified Garcia neurological function scores, indicating better neurological performance (Fig. 3A). As shown in Fig. 3B, the total brain water content in the SAH + PBS group was significantly higher than that in the Sham group and was markedly decreased after treatment with DPSC-Exos. The long-term extent of brain tissue damage was determined using HE and Nissl staining. There was more neuron damage and shrinkage morphology of neurons in the SAH + PBS group compared with the sham group. However, this phenomenon of neuronal impairment was improved in SAH + DPSC-Exos group (Fig. 3G, H).

Additionally, the Morris water maze was used to assess the rats’ spatial memory and learning abilities. The experiment was conducted 23–27 d post-SAH, including 4 d of hidden platform training and one day of consecutive probe trials. Compared with the Sham group, the SAH group showed longer escape latencies, whereas DPSC-Exos enhanced memory and spatial learning abilities (Fig. 3C–F). Moreover, in the probe trial, SAH rats treated with Exos entered the target quadrant more frequently and spent more time in the platform area than rats with SAH injected with PBS (Fig. 3E), indicating stronger memory retention.

### DPSC-Exos inhibits microglial activation and pro-inflammatory cytokine expression 24 h post-SAH

**Fig. 4:**
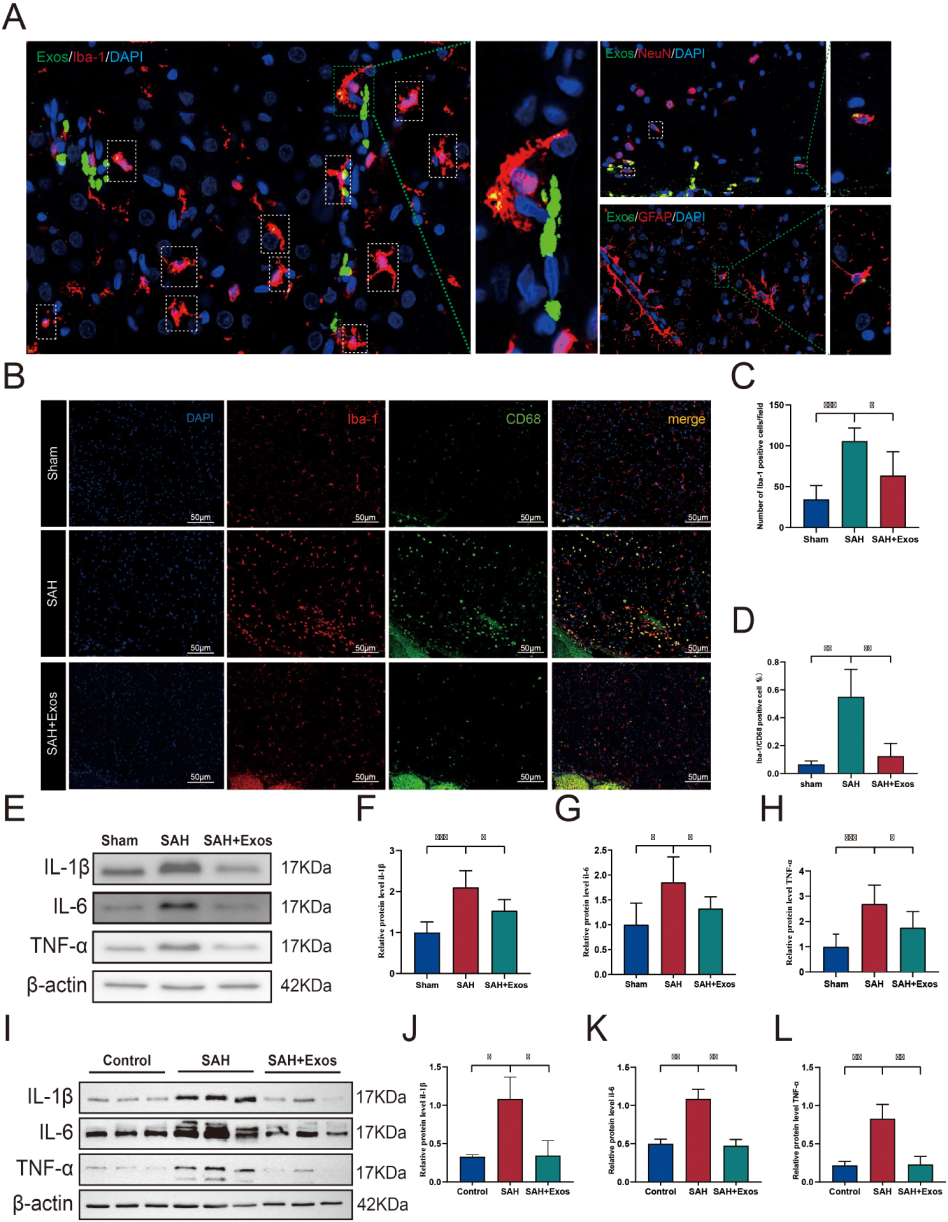
DPSC-Exos Inhibits Microglial Activation and Pro-inflammatory Cytokine Expression 24 H post-SAH. (**A**). Representative fluorescent images of brain sections stained with markers for neurons (NeuN, red), microglia (Iba-1, red), and astrocytes (GFAP, red) 24 h post-SAH. PKH67-labeled exosomes were observed entering the brains of SAH rats post intravenous administration (*n* = 2). B–D. Representative immunofluorescent micrographs (**B**) and quantitative analyses (**C**, **D**) of activated microglia (Iba-1, red) and CD68 (green) positive cells in the ipsilateral basal cortex of sham, SAH, and SAH + Exos groups. Nuclei were stained with DAPI (blue) (*n* = 4, Scale = 50 μm). (**E**–**H**). Protein levels of IL-1β, IL-6, and TNF-α in the ipsilateral hemisphere 24 h post-SAH were assessed by western blot, for each group (*n* = 6). I–L. BV2 cells treated with Hb (10 µg/mL) for 24 h and protein levels of IL-1β, IL-6, and TNF-α were assessed by western blot. Data are presented as mean ± standard deviation (SD). (*P < 0.05, **P < 0.01, ***P < 0.001) DPSC, dental pulp-derived mesenchymal stem cell; DPSC-Exo, DPSC exosome; SAH, subarachnoid hemorrhage

To study the neuroprotective mechanism of DPSC-Exos, we used an exosome tracking kit and labeled the exosomes with PKH-67. After euthanizing rats from the control and SAH groups, brain sections were prepared and immunofluorescently stained to identify microglia (Iba-1), neurons (NeuN), and astrocytes (GFAP) and to determine which cell subtype could take up exosomes. As shown in Fig. 4A, a significant number of microglia took up exosomes in the cortex around the right hemisphere hematoma.

To further clarify the response of microglia to SAH, double immunofluorescence staining for Iba-1 and CD68 was performed in the Sham, SAH + PBS, and SAH + Exos groups. Iba-1 is expressed in both resting and activated microglia, whereas CD68 is only expressed in activated microglia. In the Sham group, resting microglia stained with Iba-1 showed a typical branched morphology, whereas in the SAH + PBS group, larger activated microglia were observed (Fig. 4B). Furthermore, in the SAH rats treated with Exos, there were fewer Iba-1 positive and morphologically transformed activated microglia in the cranium (Fig. 4C). Double staining for Iba-1 and CD68 further demonstrated an increase in the number of activated microglia in the cortex surrounding the hemorrhage 24 h after SAH. Statistical analysis confirmed that the administration of Exos significantly reduced the number of Iba-1 positive and CD68 positive activated microglia (Fig. 4C, D).

To assess the activation of neuroinflammation, we used WB to measure the protein levels of pro-inflammatory factors (including IL-1β, IL-6, and TNF-α) in the right cerebral hemisphere 24 h post-SAH. Compared with the Sham group, the levels of pro-inflammatory factors significantly increased 24 h after puncture. Exo treatment inhibited the expression levels of IL-1β, IL-6, and TNF-α compared with those in the SAH + PBS group (Fig. 4E–H). To ascertain whether the increase in pro-inflammatory factors was secreted by microglia, we established an in vitro SAH model using BV2 cells. Hb (10 µM) was added to the culture medium of BV2 cells to simulate the activation of microglia, and WB was used to detect the expression levels of pro-inflammatory factors. The results showed that the Hb group exhibited increased expression levels of IL-1β, IL-6, and TNF-α. After treatment with Exos (10 µg), pro-inflammatory factor-related proteins was inhibited (Fig. 4I–L).

Therefore, our results demonstrated that DPSC-Exos can alleviate the activation of microglia and the secretion of pro-inflammatory factors 24 h post-SAH. However, the specific underlying mechanisms remain unclear.

### miRNA sequencing reveals potential key targets in DPSC-Exos

**Fig. 5:**
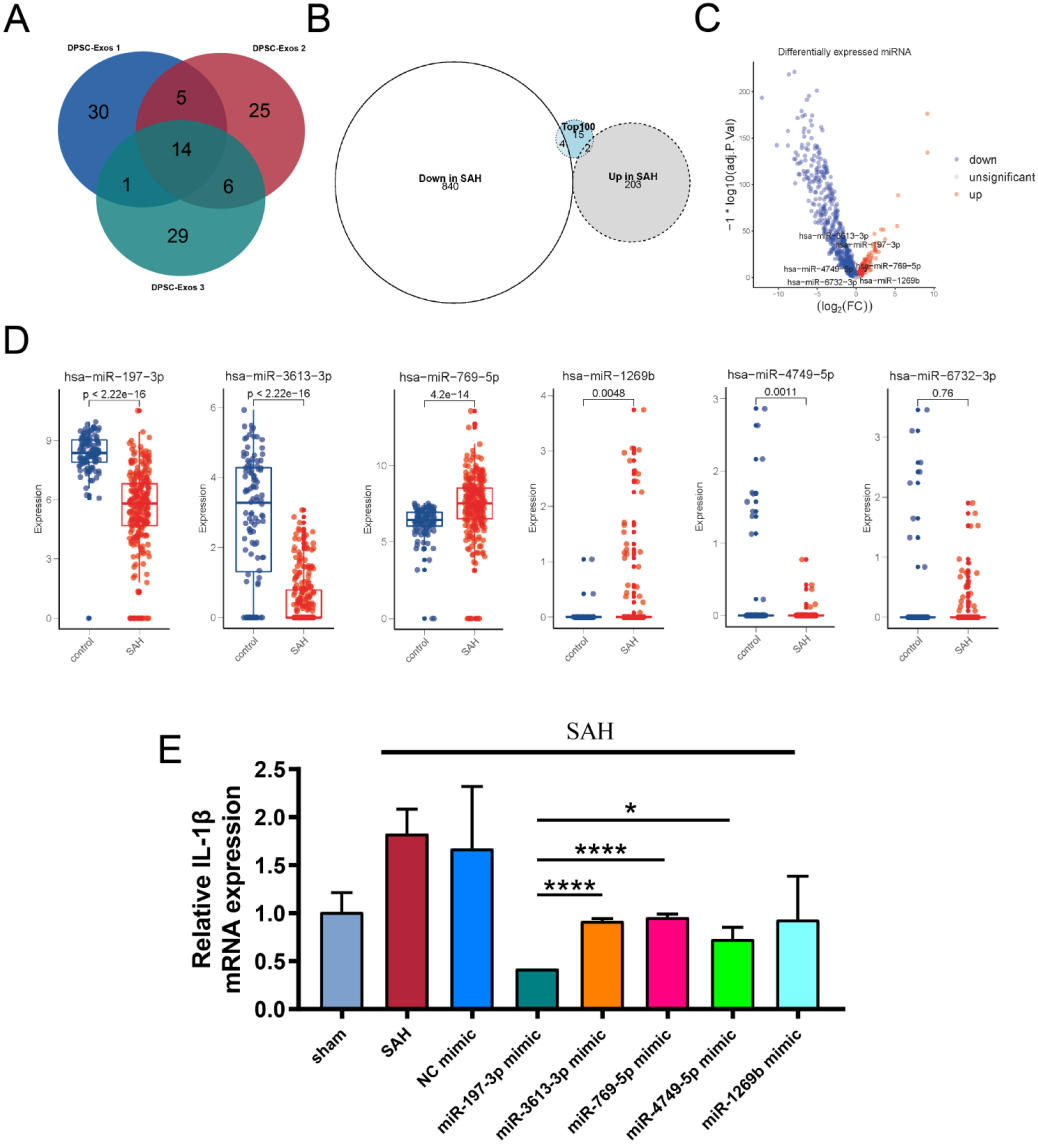
miRNA sequencing reveals potential key targets in DPSC-Exos. **A** Identification of 14 intersecting miRNAs from the top 50 miRNAs in three different biological samples using a Venn diagram. **B**–**E**. Expression changes and differences of the 14 miRNAs in cerebrospinal fluid exosomes after SAH, determined in comparison with healthy control donors. **F**. Expression of IL-1β in BV2 cells pre-treated with five differentially expressed miRNA mimics (*n* = 3). (*P < 0.05, ****P<0.0001). SAH, subarachnoid hemorrhage

MiRNA, one of the most important components of exosomes, play a significant role in exosome-mediated intercellular communication. Therefore, we employed high-throughput sequencing to identify the types and abundance of miRNAs in DPSC-Exos. To ensure biological consistency of the samples, we examined DPSC-Exos from three different biological sources. Based on the abundance of the sequencing results, we identified the top 50 miRNAs in each sample and intersected them to obtain 14 commonly expressed miRNAs (Fig. 5A).These 14 miRNAs were miR-4749-5p, miR-3665, miR-1269b, miR-7113-3p, miR-6732-3p, miR-8069, miR-4726-3p, miR-4787-5p, miR-4776-3p, miR-5093, miR-3613-3p, miR-197-3p, miR-4316, miR-6717-5p. However, according to the sequencing data (5UH2TR000891-02) from exosomes in the cerebrospinal fluid of patients with SAH obtained from the exRNA Atlas [45], we found that among the 14 significantly differentially expressed miRNAs, five miRNAs (*miRNA-197-3p*,* miRNA-3613-3p*,* miRNA-769-5p*,* miRNA-1269b*, and *miRNA-4749-5p*) showed differences between patients with SAH and healthy donors (Fig. 5B–E). However, bioinformatics analysis indicated that the concentration of miRNA-197-3p in the cerebrospinal fluid of patients with SAH was lower compared to healthy donors. Therefore, RT-qPCR was conducted to evaluate the temporal expression of *miR-197-3p* post-SAH. As shown in Figure S3, the expression level of *miR-197-3p* peaked at 12 h after SAH and then rapidly declined. Among these, *miRNA-197-3p* showed the most significant differences. We speculated that *miRNA-197-3p* may play an important role in SAH.

To further validate the regulatory role of miRNAs in neuroinflammation, BV2 cells were transfected with five selected miRNA mimics and NC (*miR-197-3p* mimic, *miR-3613-3p* mimic, *miR-769-5p* mimic, *miR-1269b* mimic, *miR-4749-5p* mimic, and miRNA NC). At 24 h after Hb stimulation, compared with other miRNAs, the *miR-197-3p* mimic group showed the lowest relative expression of *IL-1β、IL-6* and TNF-α mRNA (Fig. 5F). Based on these results, we speculated that *miR-197-3p*, which is abundant in DPSC-Exos, may be involved in the regulation of early neuroinflammation following SAH.

### The *miR-197-3p* inhibitor reverses the effects of DPSC-Exos on the downregulation of FOXO3 protein and the pyroptosis of BV2 cells

**Fig. 6:**
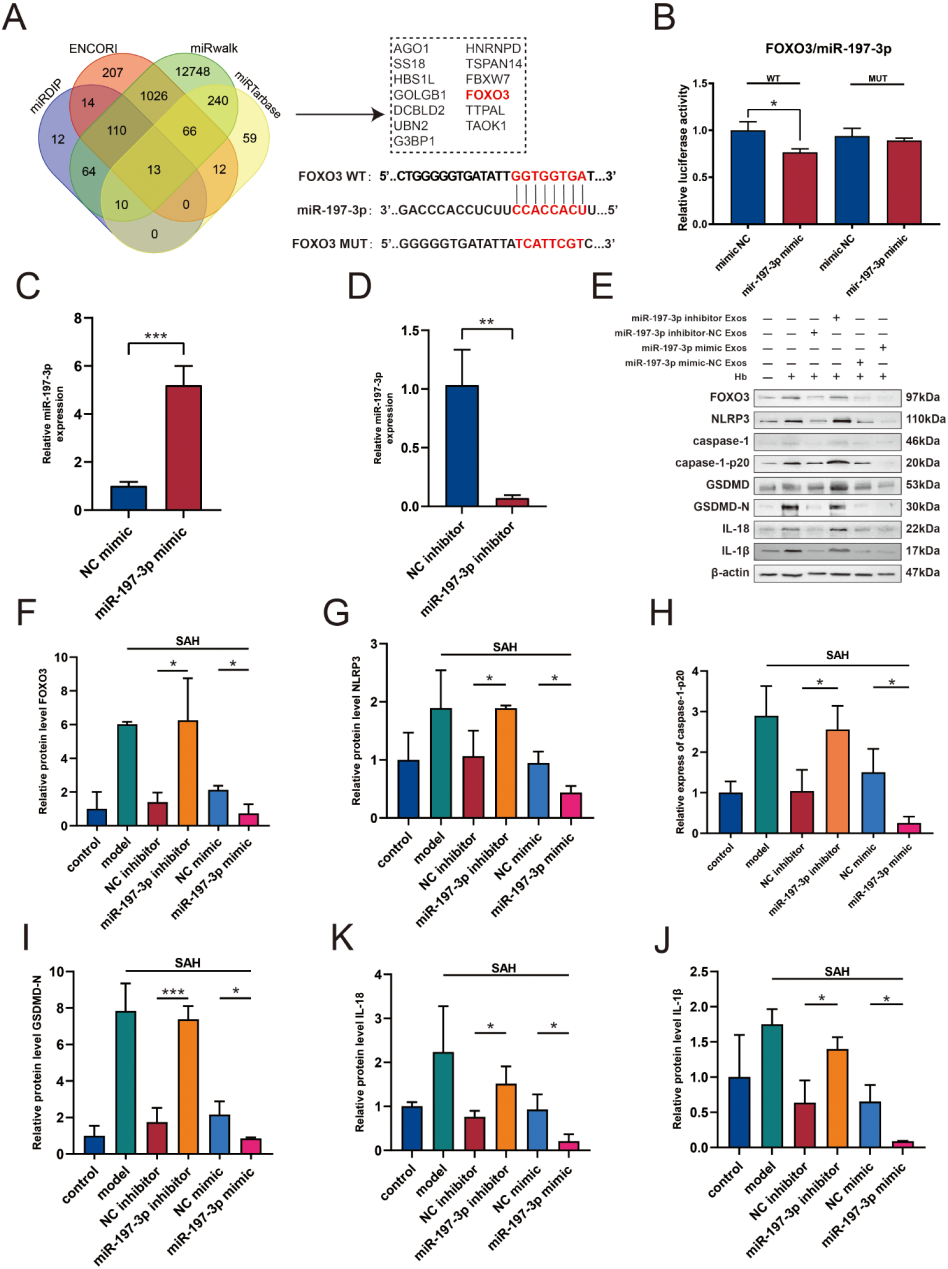
The miR-197-3p inhibitor reverses the effects of DPSC-Exos on FOXO3 and BV2 cells. **A** Prediction of potential target genes of *miR-197-3p* using four different miRNA target databases: miDIP, ENCORI, miRWalk, miRTarbase. **B**. Luciferase reporter assay validates the binding of *miR-197-3p* to the 3ʹ-UTR of *FOXO3* mRNA (*n* = 3). **C**. RT-qPCR analysis shows *miR-197-3p* mimic increases the level of *miR-197-3p* in DPSC cells (*n* = 3). **D**. RT-qPCR analysis shows *miR-197-3p* inhibitor decreases the level of *miR-197-3p* in DPSC cells (*n* = 3). **E**–**J**. Measurement of FOXO3, NLRP3, GSDMD-N, caspase1-p20, IL-18, and IL-1β protein levels by western blot in BV2 cells pre-treated with exosomes from *miR-197-3p* inhibitor or *miR-197-3p* mimic (*n* = 3). (*P < 0.05, **P < 0.01, ***P < 0.001). DPSC, dental pulp-derived mesenchymal stem cell; DPSC-Exo, DPSC exosome

MiRNAs exert their biological effects by inhibiting the expression and function of target genes [46]. Therefore, we used the public databases, miRWalk, miRDIP, miRTarbase, and ENCORI, to predict the target genes of *miR-197-3p*. *FOXO3* was identified as one of the most relevant genes (Fig. 6A). To verify whether *miR-197-3p* can bind to the 3ʹ-UTR of *FOXO3* mRNA, a dual-luciferase reporter assay was conducted. The dual-luciferase reporter assay showed that the *miR-197-3p* mimic inhibited the luciferase activity of FOXO3-WT compared with that with the mimic NC (Fig. 6B). Subsequently, the effects of *miR-197-3p* mimic and *miR-197-3p* inhibitor on DPSCs were evaluated. The RT-PCR results indicated that the *miR-197-3p* mimic increased the levels of *miR-197-3p* (Fig. 6C) and miR*-197-3p* inhibitor suppressed the levels of *miR-197-3p* (Fig. 6D). These results demonstrate the successful overexpression and knockdown of *miR-197-3p* in DPSCs.

FOXO3a is closely associated with NLRP3 inflammasome-mediated inflammatory responses [47–49]. To investigate whether DPSC-Exos could downregulate FOXO3 expression and inhibit microglial cell pyroptosis, and whether these effects were related to *miR-197-3p*, we evaluated the levels of FOXO3 and pyroptosis-related outcomes in an in vitro BV2 model. We pre-treated BV2 cells with different groups of DPSC-Exos, *miR-197-3p* mimic Exos, *miR-197-3p* mimic-NC Exos, *miR-197-3p* inhibitor Exos, and *miR-197-3p* inhibitor-NC Exos. Our results demonstrated that in the *miR-197-3p* mimic Exos group, the expression levels of NLRP3, caspase1-p20, GSDMD-N, and IL-18 were reduced, while the same expression levels increased in the *miR-197-3p* inhibitor Exos group (Figure E–J). Hoechst 33,342/PI staining showed that Hb significantly increased the number of PI-positive cells, whereas supplementation with NC-Exos and mimic-Exos significantly reduced this number (Supplementary Fig. 1), with weaker effects observed for inhibitor-Exos.

Therefore, our results indicated that DPSC-Exos inhibited hemoglobin-induced BV2 cell pyroptosis through *miR-197-3p*.

### DPSC-Exos inhibits microglial cell pyroptosis in vivo

**Fig. 7:**
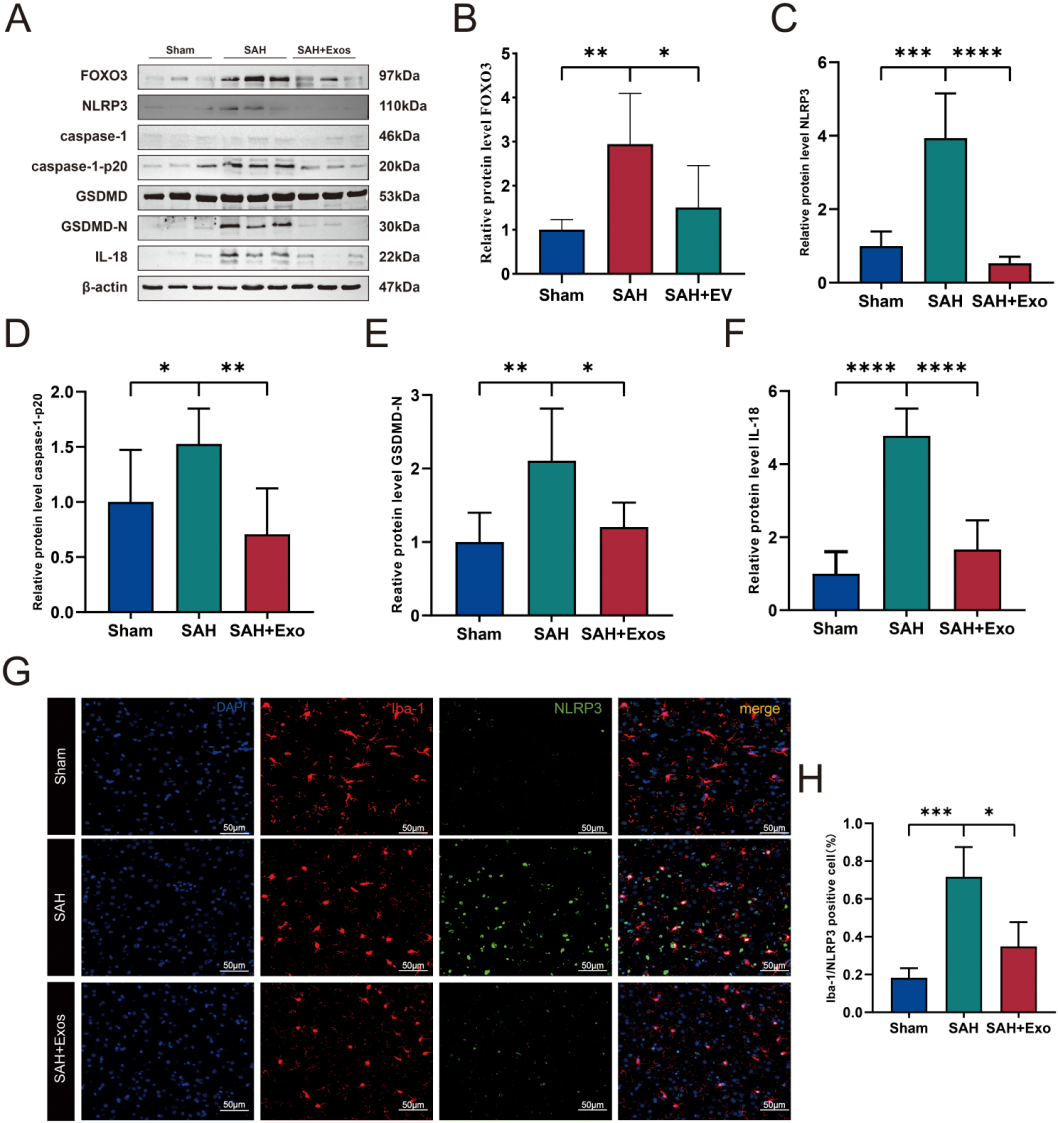
DPSC-Exos inhibits microglial cell pyroptosis by transferring miR-197-3p in vivo. **A**–**F**. Western blot analysis of FOXO3, NLRP3, GSDMD-N, caspase1-p20, and IL-1β protein expression levels in the ipsilateral cerebral hemisphere (*n* = 6). **G**, **H**. Representative immunofluorescent micrographs and quantitative analysis of microglia (Iba-1, red) and NLRP3 (green) positive cells in the ipsilateral basal cortex of Sham, SAH, and SAH + Exos groups. Nuclei stained with DAPI (blue) (*n* = 4, Scale = 50 μm). (*P < 0.05, **P < 0.01, ***P < 0.001, ****P<0.0001). DPSC, dental pulp-derived mesenchymal stem cell; DPSC-Exo, DPSC exosome; SAH, subarachnoid hemorrhage

To explore the mechanism by which DPSC-Exos alleviate neuroinflammation 24 h post-SAH in vivo, we injected DPSC-Exos into the tail vein of rats 24 h after SAH and examined the expression of related proteins (Fig. 7A). The expression of FOXO3 in the brains of rats injected with DPSC-Exos was significantly decreased (Fig. 7B). Compared with the Sham group, the expression levels of NLRP3, ASC, caspase1-p20, IL-1β, IL-18, and GSDMD-N were elevated in the SAH + PBS group, but significantly reduced after administration of DPSC-Exos (Fig. 7C–F).

To better understand the effect of DPSC-Exos on the immune response related to microglial pyroptosis after SAH, double immunofluorescence staining for NLRP3, and Iba-1 was performed. In the Sham surgery group, the cells were primarily Iba-1 positive and did not express NLRP3 (Fig. 7). After SAH, NLRP3 inflammasomes are expressed in most Iba-1 positive microglia around the ipsilateral basal cortical area. However, DPSC-Exos significantly downregulated the expression of NLRP3 (Fig. 7G). Quantitative analysis revealed that treatment with DPSC-Exos reduced the number of NLRP3 positive microglia (Fig. 7H).

### Overexpression of FOXO3 reverses the inhibitory effect of DPSC-Exo on BV2 pyroptosis

**Fig. 8:**
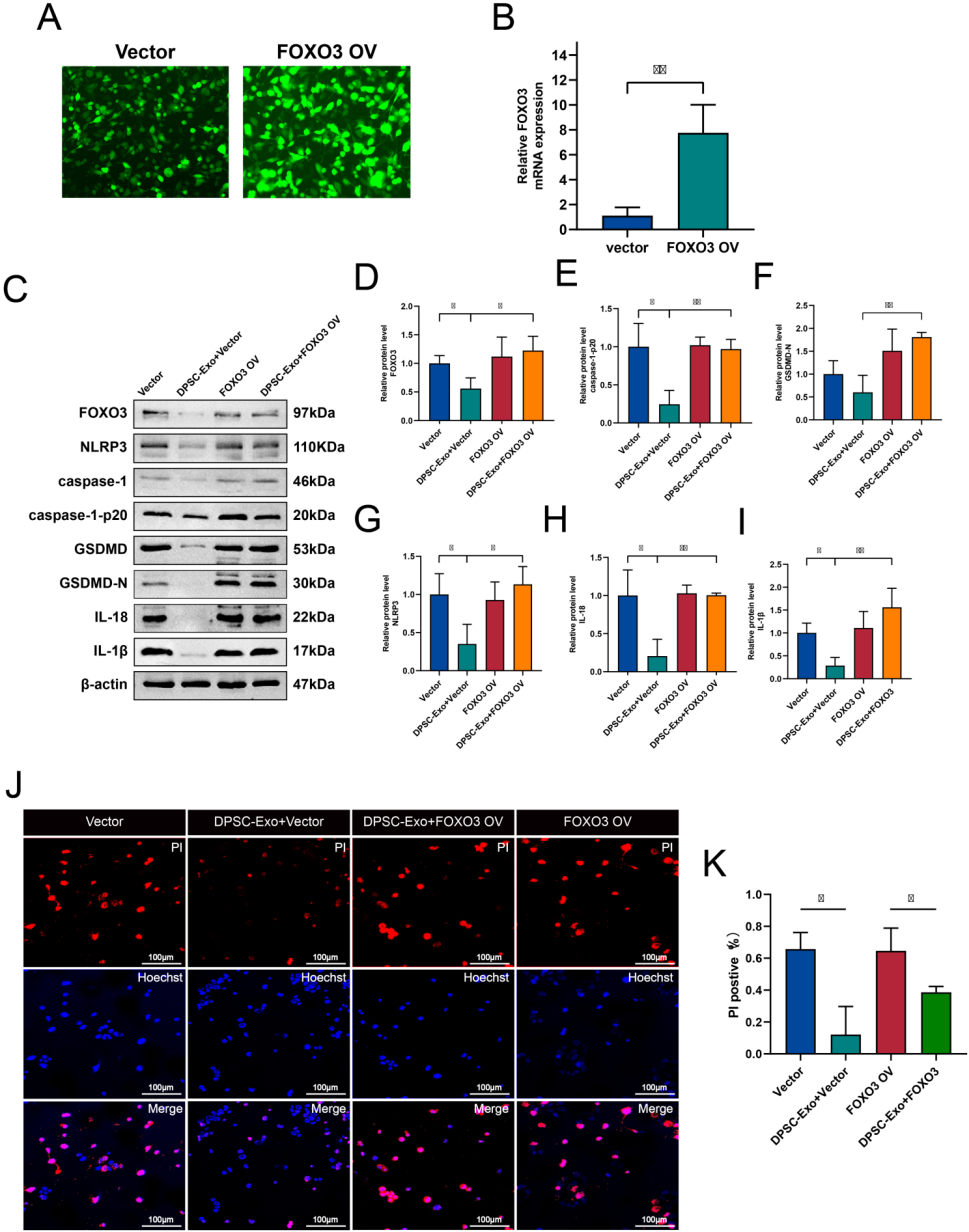
Overexpression of FOXO3 Reverses the Inhibitory Effect of DPSC-Exo on BV2 Pyroptosis (**A**) Lentiviral transfection efficiency observed under a fluorescence microscope (scale = 50 μm). (**B**) Confirmation of FOXO3 overexpression in BV2 cells by RT-qPCR (*n* = 3). **C**–**K**. BV2 cells overexpressing FOXO3 or vector co-incubated with DPSC-Exo for 24 h, then pre-treated with Hb for 24 h. Pyroptosis rate detected using Hoechst 33,342/PI staining (J, K, scale: 100 μm), and protein expression of NLPR3, caspase-1(p20), GSDMD-N, IL-18, and IL-1β analyzed by western blot (**C**– **I**). (*P < 0.05, **P < 0.01). DPSC, dental pulp-derived mesenchymal stem cell; DPSC-Exo, DPSC exosome

To investigate whether the inhibitory effect of DPSC-Exos on microglial pyroptosis was related to FOXO3, we evaluated the pyroptosis-related proteins in BV2 cells post-SAH treatment. The efficiency of lentiviral transfection was observed using fluorescence microscopy (Fig. 8A), and RT-PCR confirmed the overexpression of FOXO3 in the microglia (Fig. 8B). WB results indicated that DPSC-Exos reduced the expression levels of pyroptosis-related proteins (Fig. 8C–I) and decreased fluorescence. However, these effects were reversed by overexpression of FOXO3, suggesting that DPSC-Exos suppress hemoglobin-induced pyroptosis in BV2 cells by downregulating FOXO3 expression. Hoechst 33,342/PI staining showed that after FOXO3 overexpression, the number of PI-positive cells significantly increased, and DPSC-Exos did not reduce the number of PI-positive cells (Fig. 8J, K). These results indicate that *miR-197-3p* from DPSC-Exos can inhibit hemoglobin-induced pyroptosis in BV2 cells by downregulating FOXO3 expression.

## Discussion

Brain injury after SAH occurs in a time-dependent manner, primarily through two distinct pathological phases. The first phase is EBI, which is characterized by increased intracranial pressure, cerebral edema, global ischemia, and blood-brain barrier disruption. The second phase is delayed cerebral ischemia (DCI), which is characterized by cerebral arterial vasospasm and neuronal pyroptosis. Approximately 35% of deaths occur within 48 h post-SAH [50]. Evidence suggests that inflammatory responses play a key role in EBI, and that anti-inflammatory treatment may be crucial during the EBI phase [23]. Post-SAH activation of microglia and release of pro-inflammatory cytokines lead to neuronal damage in the cerebral cortex and hippocampus, resulting in neurological dysfunction [51]. The activation of microglia and pro-inflammatory cytokines not only damages neural cells, but also disrupts the permeability of the blood-brain barrier, leading to global cerebral edema and exacerbating secondary injury post-SAH [52]. Our results show that there is an increase in the release of pro-inflammatory cytokines IL-1, IL-6, and TNF-α in the brain at 24 h after SAH. Immunofluorescence indicated an increase in the number of pro-inflammatory Iba-1/CD68 positive microglia in the cortical area on the hemorrhagic side of the brain base. Thus, anti-inflammatory treatment for SAH may be beneficial in improving EBI associated with SAH.

A wide range of stem cell-based therapies have been applied in clinical trials, and substantial evidence supports their therapeutic efficacy [53–55]. The acquisition of MSCs, such as human BMSCs and ADSCs, typically involves invasive procedures. By contrast, DPSCs originating from the neural crest appear to be a superior source of stem cells. DPSCs can be extracted from the human wisdom or exfoliated teeth, offering a minimally invasive and safe approach without ethical concerns [56, 57]. However, the direct application of stem cells still faces potential issues, including tumorigenicity, immunosuppression, and the inability to directly cross the blood-brain barrier. MSCs exert protective effects in central nervous system diseases through paracrine mechanisms [58]. Exosomes are the most researched paracrine substances in the stem cell field. They offer several unique advantages and are being increasingly used in the treatment of neurological diseases such as stroke, intracerebral hemorrhage, and brain trauma [59–61]. Exosomes, lipid vesicles measuring 30–150 nm, can cross the blood-brain barrier and reach lesion sites [33]. They mediate intercellular communication and exert therapeutic effects by transferring functional molecules including DNA, RNA, proteins, and lipids. In this study, DPSCs were isolated and identified from extracted adult wisdom teeth. DPSC-Exos were collected from the DPSC supernatant of DPSCs using ultracentrifugation. TEM, NTA, and WB were used to characterize the DPSC-Exos. Immunofluorescence results also confirmed that PKH-67 labeled exosomes could cross the blood-brain barrier and be taken up by various cell types in the brain. These results are the first to report that DPSC-Exos can inhibit microglial activation and secretion of pro-inflammatory cytokines post-SAH and significantly alleviate global cerebral edema and neurological damage in rats.

To further investigate the molecular mechanisms and identify potential anti-inflammatory targets of DPSC-Exos in SAH, we performed whole-transcriptome sequencing and identified *miR-197-3p* as a major anti-inflammatory component of DPSC-Exos. Bioinformatics analysis of *miR-197-3p* downstream targets revealed a binding site for FOXO3, which was validated by a dual-luciferase assay. FOXO3, a member of the FOXO transcription factor family, regulates various biological processes. Downregulation of FOXO3 in SAH rats can inhibit the expression of inflammatory factors and improve short- and long-term neurological functions [52]. MiRNAs can inhibit NLRP3 expression by downregulating FOXO3, such as *miR-100-5p* in cardiomyocytes [62], *miR-30c-5p* in endothelial cells [63], and *miR-115 i*n renal tubular epithelial cells [47]. In this study, compared to the Hb group, there was a suppression in the expression of FOXO3 and pyroptosis-related proteins observed in the NC group. Upon adding miR-197-3p mimic and inhibitor, we observed a stronger regulation of FOXO3, and pyroptosis-related protein expression compared to the NC group. This highlights the significant role of miR-197-3p in regulating hemoglobin-induced cellular pyroptosis. Particularly, the overexpression of miR-197-3p, by inhibiting the expression of FOXO3, may reduce the activation of the NLRP3 inflammasome and the occurrence of pyroptosis. NLRP3-mediated neuroinflammation is related to EBI and DCI after SAH and inhibiting NLRP3 can effectively reduce brain edema and microthrombus formation and improve delayed cerebral vasospasm and functional disorders [25]. Pyroptosis is a recently reported form of the NLRP3 inflammasome-mediated cell death. Numerous studies have demonstrated a role for NLRP3-mediated pyroptosis in SAH. Targeting the NLRP3 inflammasome may improve neurological outcomes post-SAH. However, the cell type that undergoes pyroptosis in the brain is controversial. Upregulation of phosphorylated TAK1 promotes NF-κB p65 nuclear translocation and NLRP3 upregulation, inducing neuronal pyroptosis [64]. Additionally, LDC7559, a novel GSDMD inhibitor, inhibited neuronal pyroptosis post-SAH [65]. However, several studies have reported mechanisms underlying NLRP3-mediated microglial pyroptosis in SAH. One study showed that microglia-specific TREM-1 activation of the NLRP3 inflammasome triggers microglial pyroptosis [66]. Knockdown of lncRNA H19 alleviates Hb-induced primary microglial pyroptosis and NLRP3 inflammasome activation [24]. These discrepancies may be due to differences in the target protein expressing cells and the main cells of action of the drugs. Our results showed that DPSC-Exos injected intravenously were predominantly taken up by microglia. Therefore, we focused on microglial pyroptosis post-SAH. Consistent with previous studies, treatment with DPSC-Exo downregulated FOXO3, NLRP3, caspase1-p20, GSDMD-N, IL-1β, and IL-18. Immunofluorescence showed a significant reduction in NLRP3/iba-1 positive microglia in the right cerebral cortex of rats with SAH. Hence, our results suggest that DPSC-Exos inhibit microglial pyroptosis by delivering *miR-197-3p* to suppress FOXO3 expression.

## Conclusions

DPSC-Exos were successfully designed and prepared. We demonstrated that DPSC-Exos can penetrate the blood-brain barrier and be delivered to the brain. We investigated the therapeutic efficacy of DPSC-Exos in SAH rats for the first time and explored the associated molecular mechanisms. Our results indicated that DPSC-Exos can inhibit neuroinflammation and apoptosis both in vivo and in vitro. This process involves the delivery of *miR-197-3p* to microglial cells by DPSC-Exos, resulting in apoptosis and reduced *FOXO3* expression. These findings suggest that using DPSC-Exos is a promising therapeutic strategy for SAH.

### Electronic supplementary material

Below is the link to the electronic supplementary material.

Additional File 1: [Word Document -DOC/DOCX]. Supplementary Fig. 1. Pyroptosis rate in BV2 cells pre-treated with exosomes of *miR-197-3p* inhibitor or *miR-197-3p* mimic

Additional File 2: [Word Document -DOC/DOCX]. Supplementary Fig. 2. Pyroptosis rate in BV2 cells pre-treated with exosomes of *miR-197-3p* inhibitor or *miR-197-3p* mimic

Additional File 3

Additional File 4

Additional File 5

Additional File 6

Additional File 7

Additional File 8

Additional File 9

Additional File 10

Additional File 11

## Data Availability

No datasets were generated or analysed during the current study.

## Abbreviations

ADSC adipose: Derived mesenchymal stem cell
BMSC bone marrow: Derived mesenchymal stem cell
DCI: Delayed cerebral ischemia
DMEM: Dulbecco’s Modified Eagle Medium
DPSC: Dental pulp-derived mesenchymal stem cell
DPSC-Exo: DPSC exosome
EBI: Early brain injury
FBS: Fetal bovine serum
GSDMD: Gasdermin D
HE: Hematoxylin and eosin
MSC: Mesenchymal stem cell
PBS: Phosphate-buffered saline
SAH: Subarachnoid hemorrhage
WB: Western blotting
NLRP3: NOD-, LRR- and pyrin domain-containing 3
ASC: Apoptosis-associated speck-like protein containing a CARD
