# Supplementary material for: Dental pulp mesenchymal stem cell-derived exosomes inhibit neuroinflammation and microglial pyroptosis in subarachnoid hemorrhage via the miRNA-197-3p/FOXO3 axis
Source: J Nanobiotechnology. 2024 Jul 19;22:426. doi: 10.1186/s12951-024-02708-w (PMC11264715; doi:10.1186/s12951-024-02708-w)
Supplement: Supplementary file 1 — Additional File 1: [Word Document -DOC/DOCX]. Supplementary Fig. 1. Pyroptosis rate in BV2 cells pre-treated with exosomes of miR-197-3p inhibitor or miR-197-3p mimic [file 12951_2024_2708_MOESM1_ESM.docx]

**
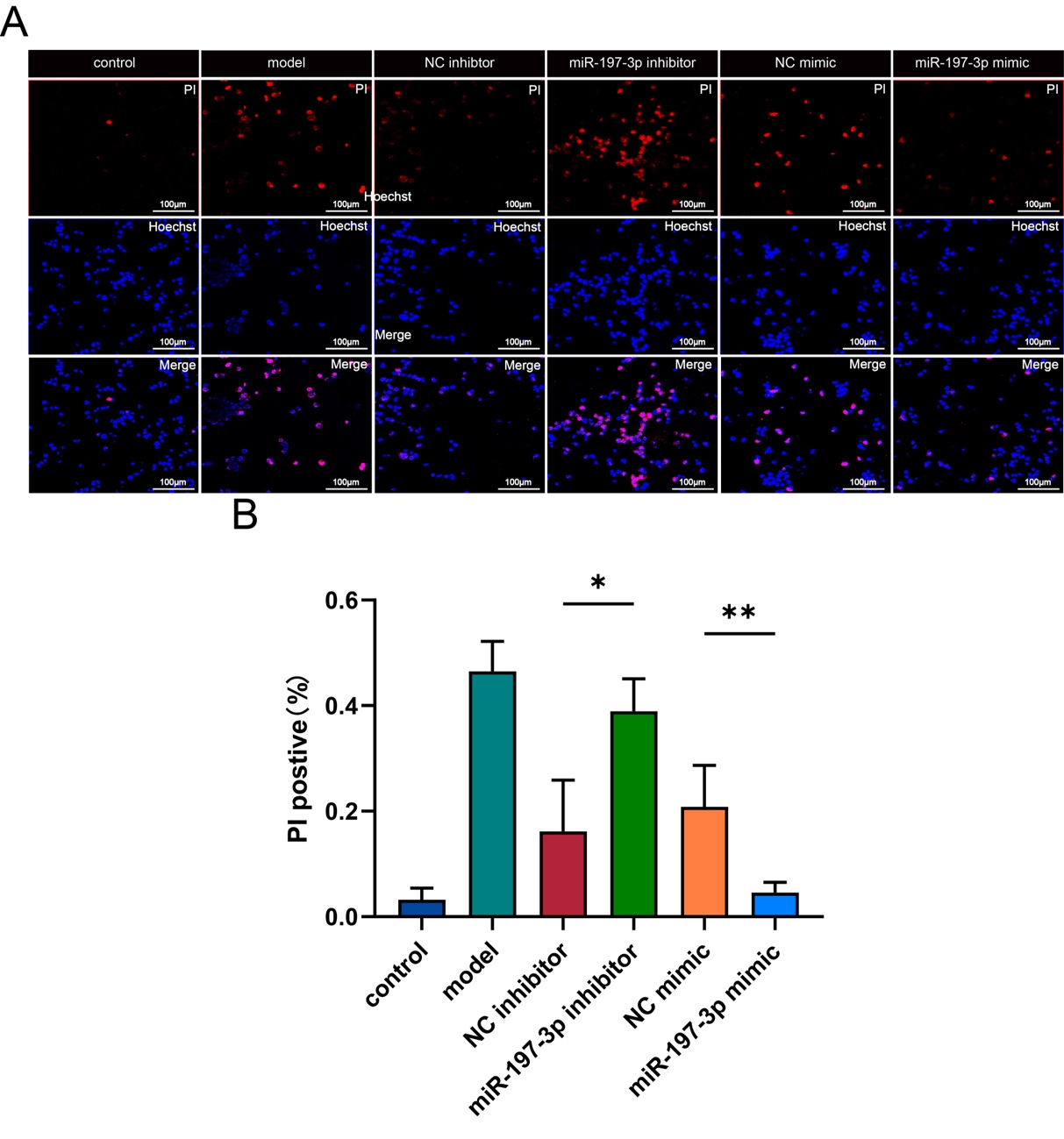
**

**Supplementary Figure 1.** Pyroptosis rate in BV2 cells pre-treated with exosomes of *miR-197-3p* inhibitor or *miR-197-3p* mimic. Images showing Hoechst 33342/PI staining. (*P<0.05, **P<0.01)
