# Supplementary material for: Dental pulp mesenchymal stem cell-derived exosomes inhibit neuroinflammation and microglial pyroptosis in subarachnoid hemorrhage via the miRNA-197-3p/FOXO3 axis
Source: J Nanobiotechnology. 2024 Jul 19;22:426. doi: 10.1186/s12951-024-02708-w (PMC11264715; doi:10.1186/s12951-024-02708-w)
Supplement: Supplementary file 4 — Additional File 4 [file 12951_2024_2708_MOESM4_ESM.docx]

Additional_File_2

Table S1 Efficiency of exosome uptake in different organs of SAH rats as demonstrated by in vivo imaging.

| Organ | Total Radiant Efficiency (p/s)/(μW/cm2) | Uptake rate (%) |
| --- | --- | --- |
| Liver | 5.65E+10 | 0.87 |
| Spleen | 3.66E+09 | 1.18 |
| Lung | 2.45E+09 | 2.03 |
| Kidney | 1.33E+09 | 3.76 |
| Brain | 7.69E+08 | 5.61 |
| Heart | 5.67E+08 | 86.55 |

Table S2 Primer for mRNA real-time polymerase chain reaction

| Genes | Primer sequence |
| --- | --- |
| BV2 |  |
| Il-6 | Forward：5’-GACTGGGGATGTCTGTAGCTC-3’ |
|  | Reverse：5’-CAACTGGATGGAAGTCTCTTGC-3’ |
| TNF-α | Forward：5’-CCCACGTCGTAGCAAACCA-3’ |
|  | Reverse：5’-ACAAGGTACAACCCATCGGC-3’ |

Figure Legend

Figure S1

Biological distribution of DPSC-Exos in vivo.

A, B. Representative IVIS images show the distribution of DiR-labeled DPSC-Exos in SAH rats 6 and 24 hours after tail vein injection (n = 2) .

C, D. Representative IVIS images demonstrate the biodistribution of DiR-labeled DPSC-Exos in six different organs including the brain, lungs, heart, spleen, liver, and kidneys, collected 6 and 24 hours after tail vein injection. These images illustrate the distribution of DPSC-Exos across these organs (n = 2) .

Figure S2 The Time course of miR-197-3p in rat brain tissue after SAH (n = 4).

Figure S3

Heatmap of the expression changes of 14 miRNAs in cerebrospinal fluid exosomes after SAH, compared to healthy control donors.

Figure S4 Chondrogenic induction of DPSCs.
A, B. Alcian blue staining used to evaluate the formation of cartilage matrix within cells (A Scale=20 μm, B Scale=10 μm).

Figure S5 ELISA was used to detect the expression of IL-1β in rat brain tissue across different dosing groups (1 μg, 10 μg, 100 μg) (n=6).

Figure S6 Expression of IL-6 and TNF-α in BV2 cells pre-treated with five differentially expressed miRNA mimics (n=3).

**Method**

**Animal**

**DPSC-Exos uptake in vivo**

To monitor the in vivo biodistribution of DPSC-Exos, the EVs were fluorescently labeled by adding 4 mg/mL of DiR (UR21017, Umibio, China) solution to PBS and incubated according to the manufacturer's instructions. The isolated Exos were then centrifuged at 140,000 g for 1 hour at 4°C, and the pellet was resuspended in PBS.100 μg of DiL-labeled Exos were injected into the tail vein of SAH model rats. 6 or 24 hours later, the rats were anesthetized, and images of the DPSC-Exos distribution in vivo were taken using a live imaging system (IVIS Lumina XRNS III; Perkin Elmer, Germany). Additionally, organs including the brain, lungs, heart, spleen, liver, and kidneys were collected at the corresponding time points and labeled Exos were quantified ex situ using the same imaging system.

**The time course of miR-197-3p in rat brain tissue after SAH.**

32 rats were randomly divided into eight groups (n=4 per group) to establish a time-gradient animal model of SAH. The groups included: Sham, 3 hours, 6 hours, 12 hours, 24 hours, 48 hours, 72 hours, and 1 week. Rats were euthanized at different time points post-SAH, and the expression trend of miRNA-197-3p in brain tissue after SAH was determined using RT-qPCR.

**Chondrogenic induction of DPSCs.**

DPSCs (2.5×10^5^ cells per well) were seeded in a six-well plate. After 21 days of culture in chondrogenic differentiation medium (complete medium supplemented with 0.1 μM dexamethasone, 40 μg/mL L-proline, 10 μg/mL insulin-transferrin-selenium-sodium pyruvate, 50 μg/mL ascorbate-2-phosphate, and 10 ng/mL transforming growth factor-β3), the multipotent differentiation ability of DPSCs was evaluated by Alcian Blue staining.

**ELISA was used to detect the expression of IL-1β in different dosing groups.**

24 hours after the occurrence of subarachnoid hemorrhage (SAH), rats were deeply anesthetized and euthanized by transcardial perfusion with ice-cold PBS. The brain tissue was dissected into ipsilateral and contralateral hemispheres. The brain tissue was homogenized, and then the homogenate was centrifuged at 14,000 × g for 30 minutes. The supernatant was collected for subsequent experiments. The concentration of IL-1β in the brain tissue lysates was analyzed using a commercial ELISA kit (ER1567, FineTest, China) following the manufacturer's instructions. The final concentration of the cytokine was determined based on the standard curve of absorbance.
