# Supplementary figures and images for: Dental pulp mesenchymal stem cell-derived exosomes inhibit neuroinflammation and microglial pyroptosis in subarachnoid hemorrhage via the miRNA-197-3p/FOXO3 axis
Source: J Nanobiotechnology. 2024 Jul 19;22:426. doi: 10.1186/s12951-024-02708-w (PMC11264715; doi:10.1186/s12951-024-02708-w)

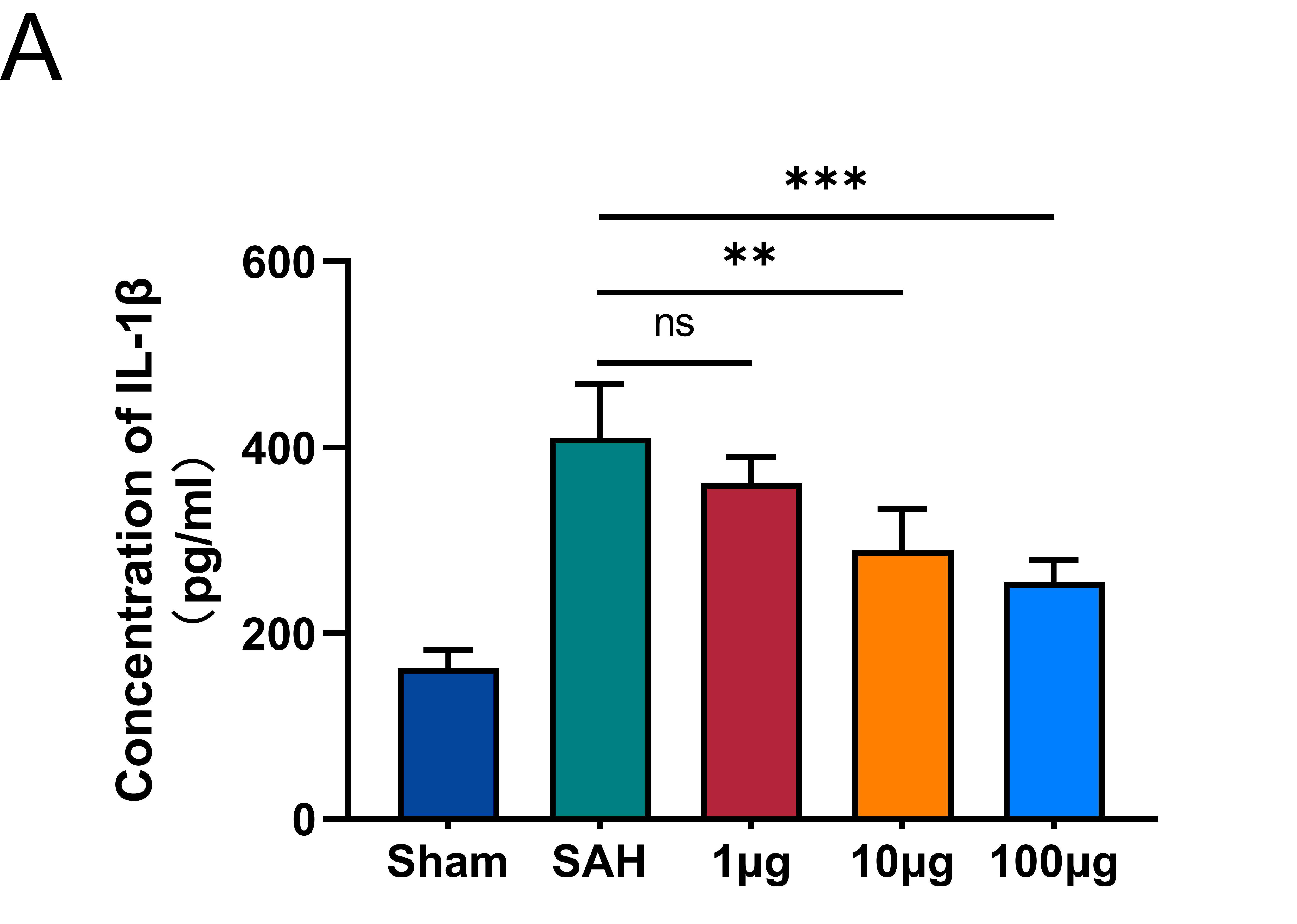

Supplement: Supplementary file 5 — Additional File 5 [file 12951_2024_2708_MOESM5_ESM.jpg]

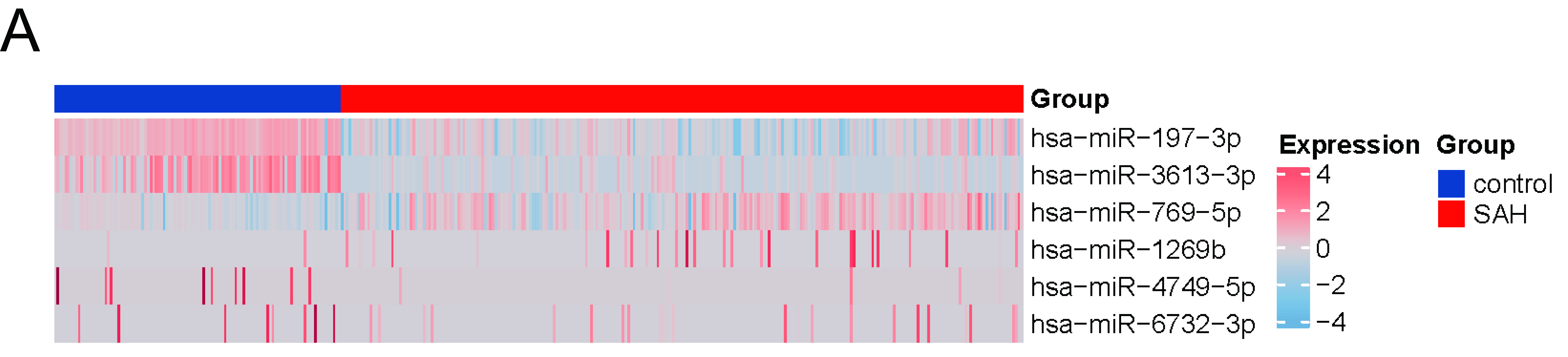

Supplement: Supplementary file 6 — Additional File 6 [file 12951_2024_2708_MOESM6_ESM.jpg]

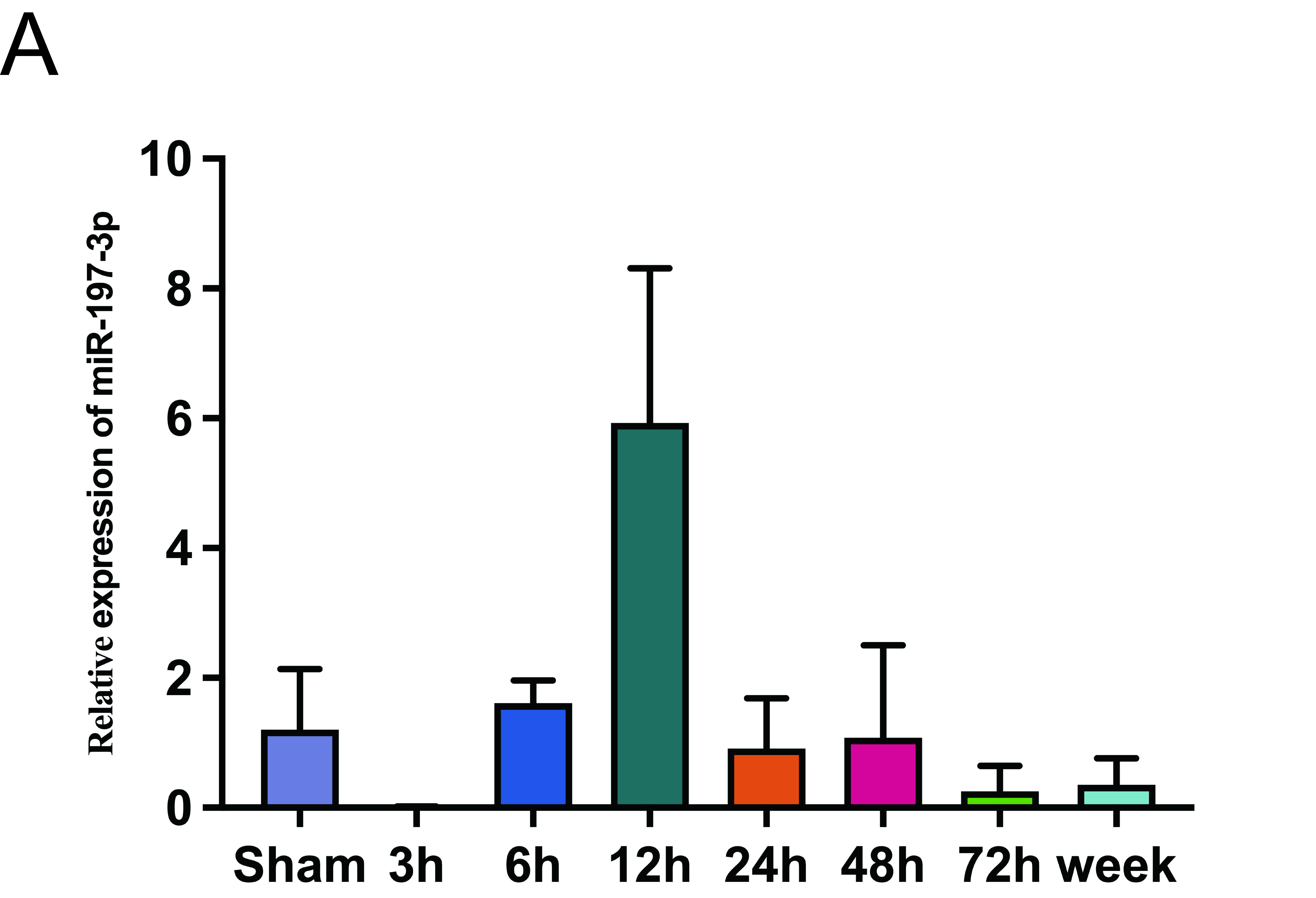

Supplement: Supplementary file 7 — Additional File 7 [file 12951_2024_2708_MOESM7_ESM.jpg]

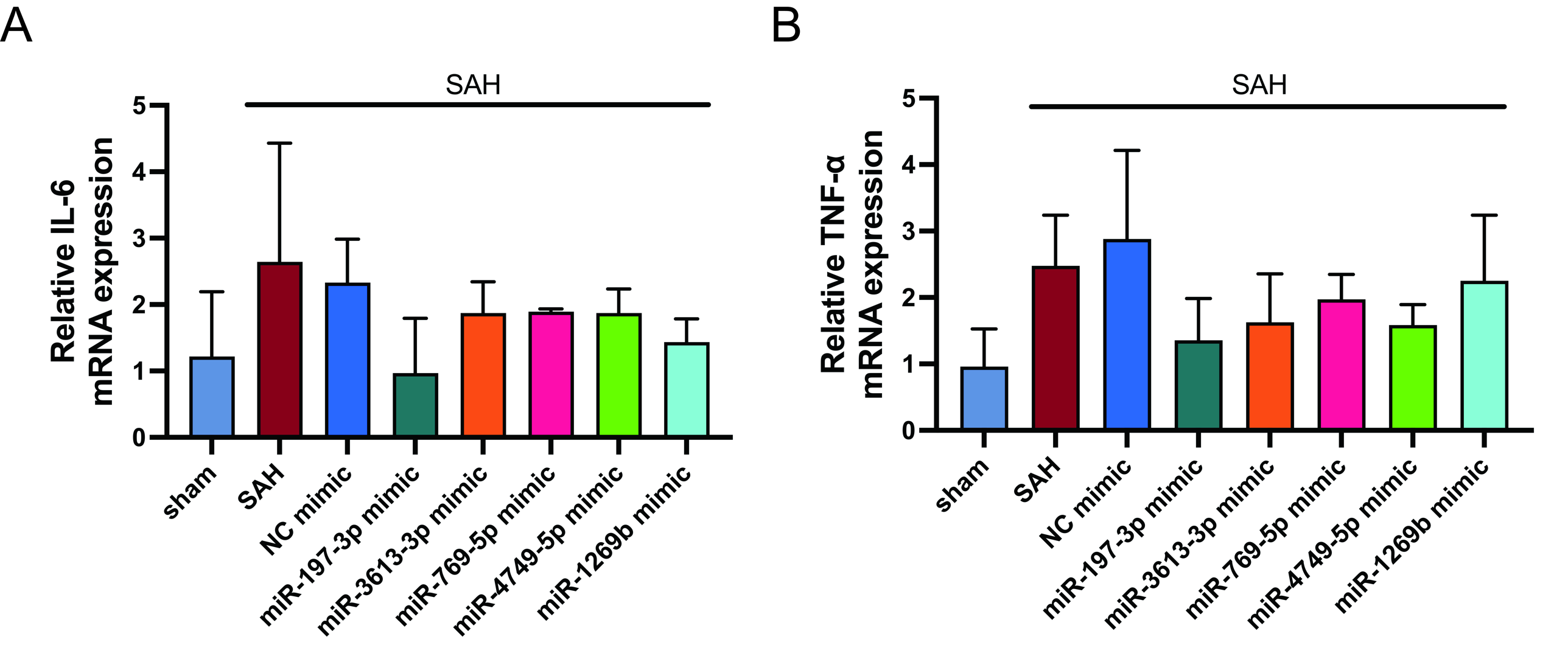

Supplement: Supplementary file 8 — Additional File 8 [file 12951_2024_2708_MOESM8_ESM.jpg]

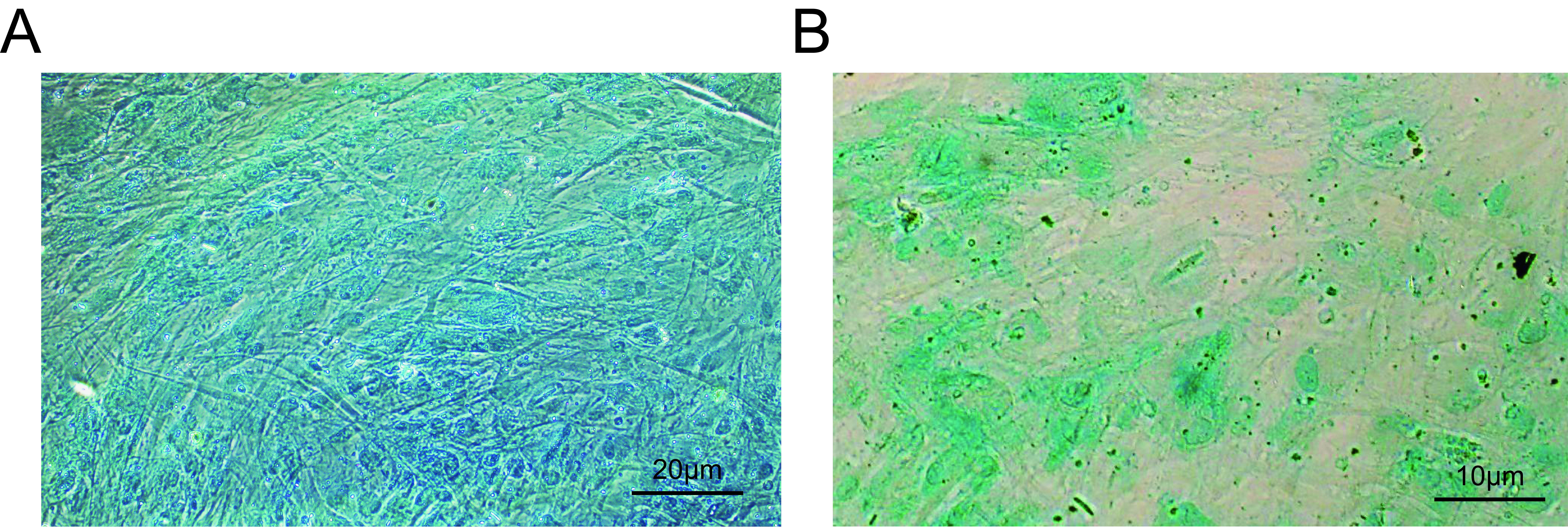

Supplement: Supplementary file 9 — Additional File 9 [file 12951_2024_2708_MOESM9_ESM.jpg]

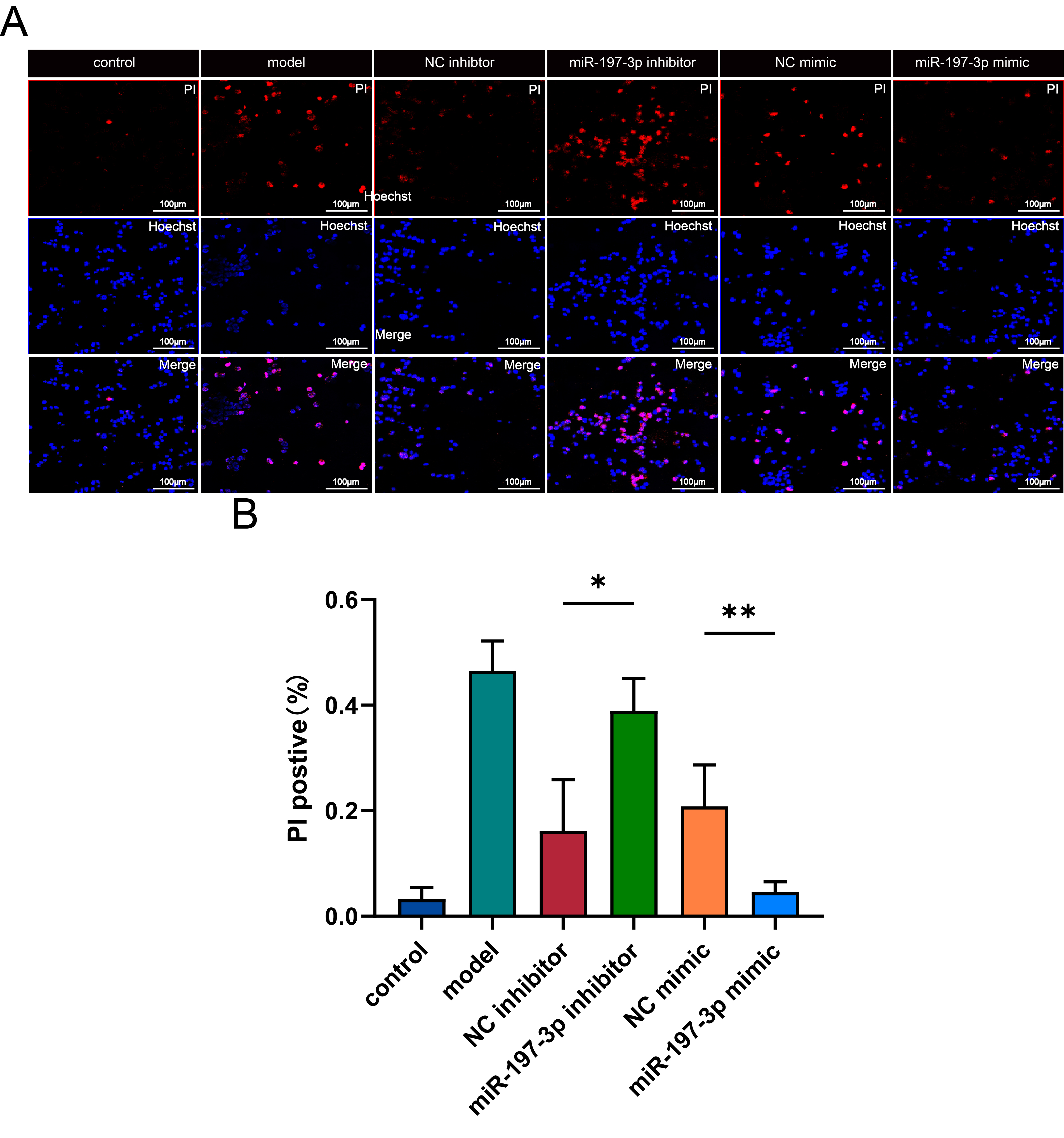

Supplement: Supplementary file 11 — Additional File 11 [file 12951_2024_2708_MOESM11_ESM.jpg]
